# Thallium(I) Tropolonates: Synthesis, Structure, Spectral Characteristics, and Antimicrobial Activity Compared to Lead(II) and Bismuth(III) Analogues

**DOI:** 10.3390/molecules27010183

**Published:** 2021-12-29

**Authors:** Krzysztof Lyczko, Monika Lyczko, Marzena Banasiewicz, Karolina Wegrzynska, Anna Ziółko, Anna Baraniak, Jan Cz. Dobrowolski

**Affiliations:** 1 Institute of Nuclear Chemistry and Technology, Dorodna 16, 03-195 Warsaw, Poland; m.lyczko@ichtj.waw.pl (M.L.); j.dobrowolski@nil.gov.pl (J.C.D.); 2 Institute of Physics PAS, Al. Lotnikow 32/46, 02-668 Warsaw, Poland; mbanas@ifpan.edu.pl; 3 National Medicines Institute, Chełmska 30/34, 00-725 Warsaw, Poland; k.wegrzynska@nil.gov.pl (K.W.); a.ziolko@nil.gov.pl (A.Z.); a.baraniak@nil.gov.pl (A.B.)

**Keywords:** thallium(I) triflate, thallium(I) complexes, tropolone, 5-methyltropolone, hinokitiol, crystal structure, antimicrobial activity

## Abstract

Synthesis, single-crystal X-ray determination diffraction and FT-IR, NMR (^1^H, ^13^C, ^19^F and ^205^Tl), UV–vis, and luminescence spectra characteristics were described for series of thallium(I) compounds: thallium(I) triflate (Tl(OTf)), 1:1 co-crystals of thallium(I) triflate and tropolone (Htrop), Tl(OTf)·Htrop, as well as simple thallium(I) chelates: Tl(trop) (**1**), Tl(5-metrop) (**2**), Tl(hino) (**3**), with Htrop, 5-methyltropolone (5-meHtrop), 4-isopropyltropolone (hinokitiol, Hhino), respectively, and additionally more complex {Tl@[Tl(hino)]_6_}(OTf) (**4**) compound. Comparison of their antimicrobial activity with selected lead(II) and bismuth(III) analogs and free ligands showed that only bismuth(III) complexes demonstrated significant antimicrobial activity, from two- to fivefold larger than the free ligands.

## 1. Introduction

Tropolone (2-hydroxy-2,4,6-cycloheptatriene-1-one, Htrop) and its derivatives contain the aromatic seven-membered ring with the neighboring carbonyl and hydroxyl groups. They exhibit interesting physicochemical properties, as well as antitumor, insecticidal, fungicidal, antimicrobial, and neuroprotective activity [1,2,3,4,5,6]. Moreover, they are also suitable chelators for various metal ions [7,8]. Due to their broad and potent antimicrobial activity, some naturally occurring tropolones are components of the formulations for cosmetics and dentistry [9,10].

The first tropolonato complexes with the *p*-block, period-6, metal ions, such as [Bi^III^(trop)_2_Cl], Na[Bi^III^(trop)_4_], Bi^III^(trop)_3_, Pb^II^(trop)_2_, and Pb^IV^(trop)_4_, were synthesized more than half a century ago [11,12]. In the 1990s, a new series of bismuth(III) complexes with tropolones was synthesized; however, the structures of only two of them, aquabis(4,5-benzotropolonato)bismuth(III) nitrate and nitratobis(tropolonato)bismuth(III), were determined [13]. Over a decade ago, we demonstrated that formation of different polymeric or dimeric lead(II) tropolonates ([Pb_3_(trop)_4_(ClO_4_)_2_]_n_, [Pb_2_(trop)_2_(NO_3_)_2_MeOH]_n_, [Pb(trop)(CF_3_SO_3_)(H_2_O)]_n_, and [Pb(trop)_2_]_2_) depended on pH and counterion presence [14]. A few years ago, we also performed a similar study on bismuth(III) tropolonates and 5-methyltropolonates (5-meHtrop): ([Bi_2_(trop)_4_(ClO_4_)_2_]_n_, [Bi_2_(trop)_4_(NO_3_)_2_]_n_, [Bi(trop)_2_(CF_3_SO_3_)]_2_, {[Bi_2_(5-metrop)_4_(ClO_4_)MeOH]ClO_4_}_n_, Bi(5-metrop)_2_(NO_3_) and [Bi_2_(5-metrop)_4_(CF_3_SO_3_)_2_]_n_) [15]. Recently, we revealed that in the [Pb(trop)_2_(Htrop)] and [Bi(trop)_2_(Htrop)(CF_3_SO_3_)] structures, the tropolone ligands act as both a neutral monodentate ligand (Htrop) and a bidentate chelating anion (trop^−^) [16]. For 4-isopropyltropolone (hinokitiol, Hhino) the simple [Pb(hino)_2_]_n_ and Bi(hino)_3_EtOH complexes have only been presented [7,17].

Despite many studies on the heaviest *p*-block metal tropolonates, no thallium(I) analogues have been presented until now, except structural characterization of two, the Tl^III^(trop)Me_2_ and Tl^III^(trop)Ph_2_ complexes [18,19].

A rich structural variety of thallium(I) compounds [20,21,22,23,24,25,26,27,28] makes them interesting for structural and materials research. The diversity stems from the possibility of the Tl^+^ ion to adopt a wide range of coordination numbers, and the presence of 6*s*^2^ electrons enabling participation in Tl∙∙∙X (X–Tl, C, H or halogen atoms) secondary interactions. In addition, attention recently has been focused on thallium(I) compounds bearing metallophilic interactions with other metal centers [29,30,31,32]. Still, unlike most *p*-block metals, the thallium compounds are infrequently studied due to their high toxicity. Taking all the above into account and being inspired by earlier works on lead(II) and bismuth(III) tropolonates, here, we characterized structures of new thallium(I) compounds with unsubstituted tropolone, 5-methyltropolone, and hinokitiol. Moreover, after sustained efforts, we obtained thallium(I) triflate single crystals appropriate for the X-ray determination. Eventually, we examined the in vitro antimicrobial activity of new thallium(I) complexes and free tropolone ligands, and compared it with those of similar lead(II) and bismuth(III) complexes we studied previously [14,15,17]. Various metal complexes are still interesting due to their potential antibacterial properties [33,34,35,36].

## 2. Results and Discussion

### 2.1. Synthesis

The thallium(I) tropolonate complexes cannot be obtained by simple mixing of thallium(I) nitrate(V) or triflate with a tropolone ligand. An attempt of such a direct synthesis ends with 1:1 (Tl(OTf)·Htrop) co-crystals. Moreover, similar adducts with the other tropolone derivatives are not formed. Simple thallium(I) complexes with tropolone (**1**) and 5-methyltropolone (**2**) can be synthesized by adding equimolar amounts of ammonia to the mixture of thallium(I) nitrate(V) or triflate salt and the respective tropolone derivative. A simple Tl(hino) complex (**3**) is formed only when using thallium(I) nitrate(V), while in the case of the triflate salt, the more complex compound {Tl@[Tl(hino)]_6_}(OTf) (**4**) is obtained.

### 2.2. Crystal Structures

Crystal structures of all the thallium(I) compounds were determined at low temperature using a single-crystal X-ray diffraction method (Supplementary Materials Figure S1).

#### 2.2.1. Crystal Structures of Thallium(I) Triflate and Its Adduct with Tropolone

**Figure 1:**
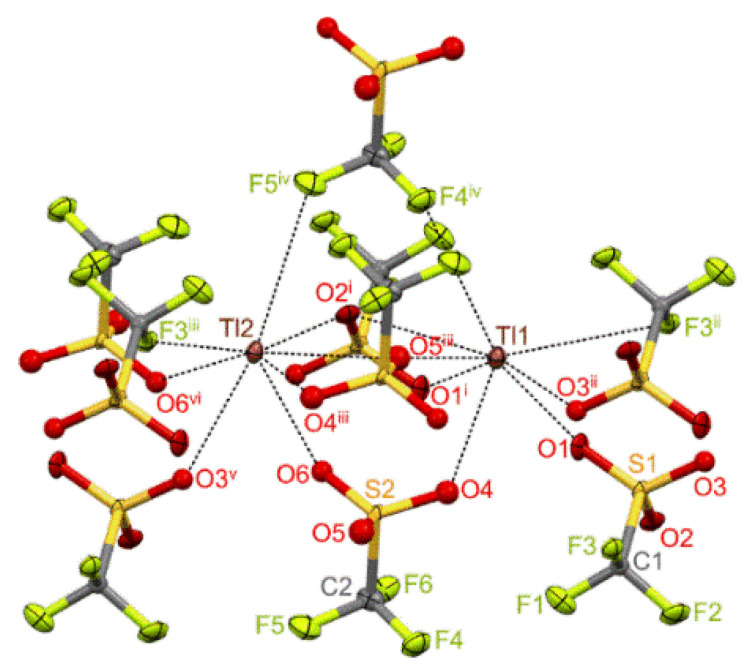
A fragment of the crystal structure of thallium(I) triflate showing the near surroundings around thallium(I) ions (atoms, except for O3–O6 and C1, shown as 50% ellipsoids).

Despite that the thallium(I) triflate (Tl(OTf)) was mainly amorphous, it was possible to obtain some crystalline material suitable enough for scXRD measurements. As a result, it was found that two crystallographically independent thallium(I) ions were present in the crystal structure of Tl(OTf) (Figure 1). Both of them were surrounded by six oxygen atoms from five neighboring triflate ions, with an average Tl···O distance of 2.88(2) Å for Tl1 (range 2.72(2)–3.09(2)) and 2.89(2) Å for Tl2 (range 2.78(2)–3.06(2)). In addition, two Tl···F contacts from two additional triflate ions in the range of 3.22(1)–3.43(1) Å, which were close to the sum of the van der Waals radii of Tl and F atoms (3.43 Å), were found for both metal ions [37]. All these Tl···O and Tl···F distances are shown in Table 1. The neighboring thallium(I) cations remained at distances of approximately 4.35(1) (Tl2···Tl2), 4.62(2) (Tl1···Tl1), 4.60(2), and 4.66(2) Å (Tl1···Tl2) from each other.

**Figure 2:**
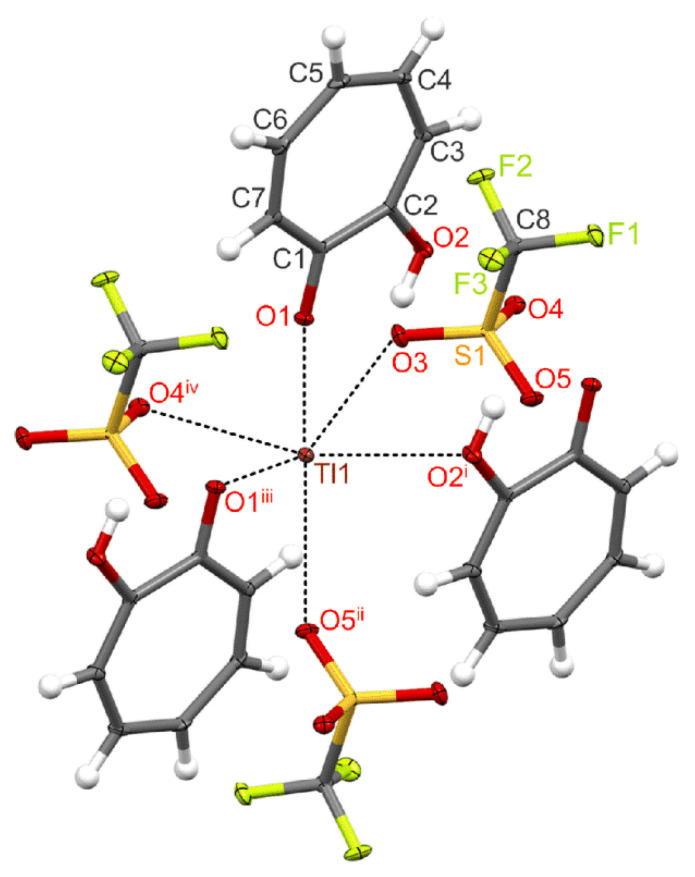
A fragment of the crystal structure of Tl(OTf)·Htrop adduct showing the near surroundings around the thallium(I) ion (nonhydrogen atoms shown as 50% ellipsoids).

The structure of the titled adduct consisted of thallium(I) triflate and tropolone at a molar ratio of 1:1. Each metal ion was surrounded by three tropolone molecules and three triflate anions. Each triflate and tropolone parts was connected with three thallium(I) ions. The metal ion in this structure was surrounded by six oxygen atoms (Figure 2). Three of them belonged to the three triflate groups. There were also three oxygen atoms from three tropolone molecules, including two from carbonyl groups and one from the hydroxyl group. The Tl···O distances were in the range of 2.722–3.040(2) Å (Table 1). The shortest metal–oxygen contacts, 2.722(2) (Tl1···O1) and 2.744(2) Å (Tl1···O1^i^), were those with carbonyl groups of tropolone molecules. Slightly longer Tl–O bonds of 2.871(2) (Tl1···O4^ii^), 2.934(2) (Tl1···O5^iii^), and 2.942(2) Å (Tl1···O3) from the CF_3_SO_3_^−^ ions, and 3.040(2) Å (Tl1···O2^iv^) from the OH group of tropolone, completed the coordination sphere around thallium(I). These thallium–oxygen distances were much shorter than the calculated sum of the van der Waals radii of thallium and oxygen atoms (3.48 Å [37]). The arrangement of these six oxygen atoms around the metal center formed a strongly distorted octahedron (or distorted triangle antiprism). The presented structure may be considered to contain Tl^+^ cations embedded within a network completed by the triflate anions and tropolone molecules. The presence of relatively weak Tl···O interactions suggested that this network was essentially ionic.

In the Tl(OTf)·Htrop structure, the tropolone molecules were not involved in the formation of O–H∙∙∙O hydrogen-bonded dimers, as they were in the case of pure tropolone [38]. However, it was stabilized by intermolecular O2–H2∙∙∙O4 hydrogen bonds of 2.941(3) Å formed between tropolone molecules and triflate anions.

In the Cambridge Structural Database, there are several crystal structures that include thallium(I) triflate as a part of the structure [26,39,40,41,42,43,44,45,46,47,48,49,50,51].

#### 2.2.2. Complexes with Tropolone and 5-Methyltropolone: Tl(trop) (**1**) and Tl(5-metrop) (**2**)

**Figure 3:**
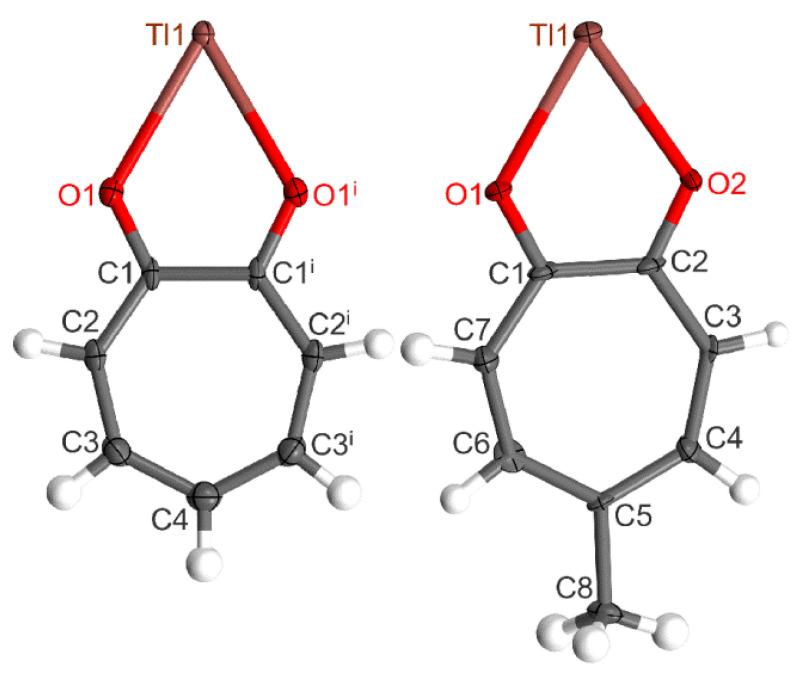
Molecular structures of Tl(trop) (**1**) (left) and Tl(5-metrop) (**2**) (right) (symmetry code: (i) −*x* + 1, *y*, −*z* + 1.5). Nonhydrogen atoms are shown as 50% ellipsoids.

The molecular structures of the studied complexes with tropolone and its derivative substituted in position 5 by the methyl group are presented in Figure 3, and their selected bond parameters are listed in Table 2. The asymmetric units of the presented structures consisted of either a half or one molecule of the thallium(I) complex in **1** or **2**, respectively. The crystal structures of these two compounds consisted of mononuclear Tl(trop) or Tl(5-metrop) units in which tropolones behaved as anionic bidentate ligands. Each thallium(I) ion was chelated by one ligand, resulting in a mean Tl–O bond length of 2.472(9) Å in **2** and 2.588(3) Å in **1**. The bite angles, formed within five-membered chelate rings, equaled 61.1(1) and 65.7(3)° for **1** and **2**, respectively. The C–O bonds in the range of 1.28–1.30 Å were distinguished within each ligand moiety. Thus, these distances were intermediate between those observed for double C=O (*ca.* 1.25 Å) and single C–O (*ca.* 1.34 Å) bonds in the free tropolone and 5-methyltropolone molecules [38,52].

**Figure 4:**
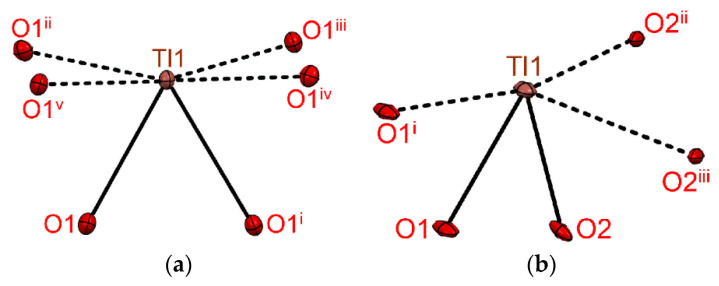
Coordination spheres of thallium(I) ions in complexes: (**a**) **1** and (**b**) **2** (symmetry codes as in Table 2). Solid and dashed lines indicate chelating bonds and intermolecular contacts, respectively.

**Figure 5:**
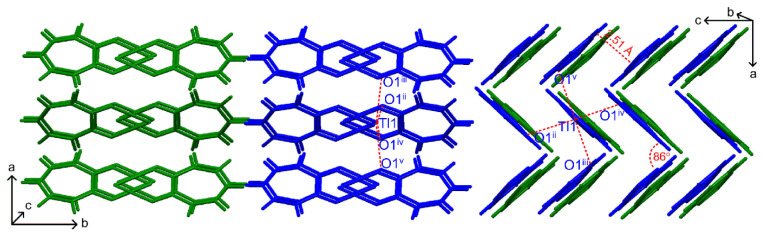
Two different views of the arrangement of Tl(trop) chelates in the extended structure of **1** showing the chosen intermolecular Tl···O contacts.

Coordination environments around chelated thallium(I) ions in the studied complexes, as shown in Figure 4, were completed by three or four additional oxygen atoms from neighboring molecules. These thallium–oxygen contacts were much shorter than the calculated sum of their van der Waals radii (3.48 Å [37]). The crystal structure of **1** was stabilized by a connection of the Tl(trop) units into layers (Figure 5), with two types of bridging Tl···O distances of 2.906(3) and 2.947(3) Å between adjacent molecules. The aromatic rings were directed outside of these layers. The molecules of **1** could also be considered as placed in planes intersecting at an angle of approximately 86° with distances of 2.51 Å between them.

**Figure 6:**
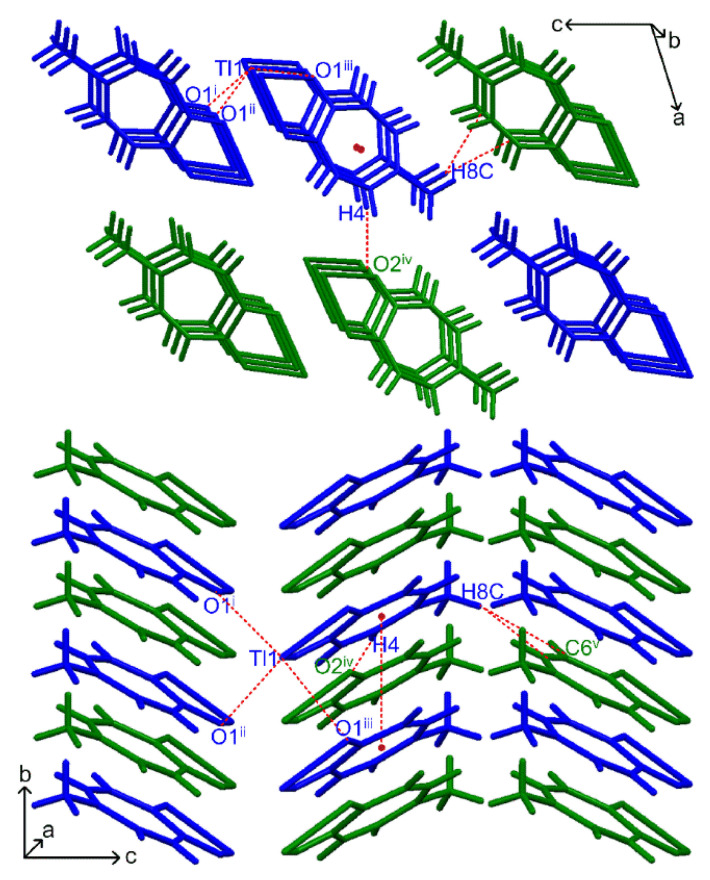
Two different views of the arrangement of Tl(5-metrop) chelates in the extended structure of **2** showing the chosen intermolecular contacts.

The crystal structure of **2** could be described as a connection of chelate units into chains extending along the *b*-axis (Figure 6). Within two adjacent chains the molecules of **2** were held together by bridging thallium–oxygen contacts of 2.873(10), 2.888(10), and 3.189(9) Å, clearly longer than those observed for the chelating bonds. In addition, these pairs of chains were stabilized together by the presence of weak intermolecular hydrogen bonds of 3.35(1) (C4–H4···O1), 3.90(1) (C8–H8C···C6), and 4.01(2) Å (C8–H8C···C7). Moreover, in contrast to **1**, the crystal structure of the complex with 5-methyltropolonato ligand was stabilized through intermolecular π···π interactions between neighboring aromatic rings, with distances of 3.84 Å between their centroids. In addition, the shortest distance between metal atoms in **2** (3.843(2) Å) was a bit less than twice the van der Waals radius of thallium (3.92 Å [37]), and could be considered as a weak metallophilic bonding. This distance was shorter than the analogous contacts in **1** (4.1822(2) and 4.1926(2) Å). It was noteworthy that in contrast to **1**, the complex with 5-methyltropolone had a nonplanar chelate ring with the Tl atom lying about 0.38 Å out of plane formed by the remaining atoms. It is worth adding that only a few complexes of 5-methyltropolone with bismuth(III) ion have been structurally characterized until now [15].

#### 2.2.3. Complexes with Hinokitiol: Tl(hino) (**3**) and {Tl@[Tl(hino)]_6_}(OTf) (**4**)

**Figure 7:**
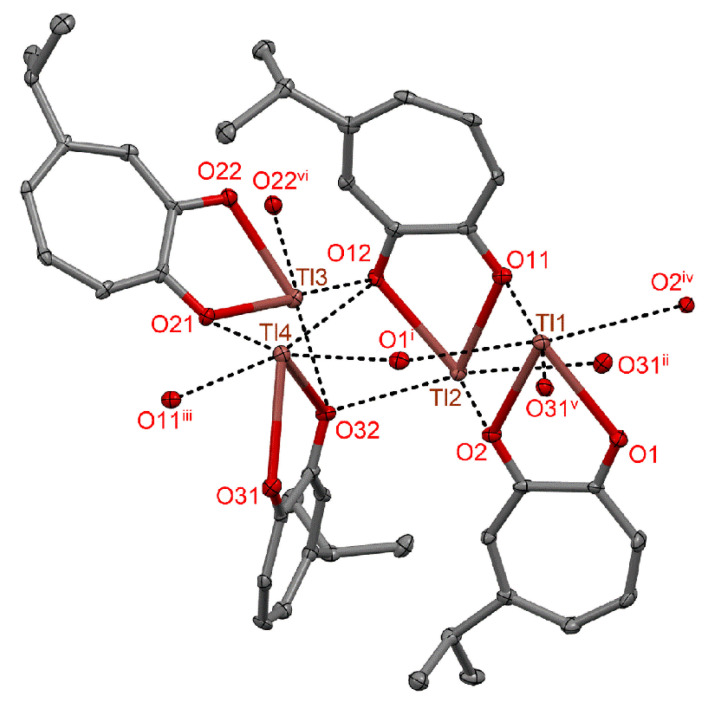
Asymmetric unit of **3** with intermolecular contacts between Tl(hino) molecules (atoms shown as 50% ellipsoids; hydrogen atoms were omitted for clarity).

**Figure 8:**
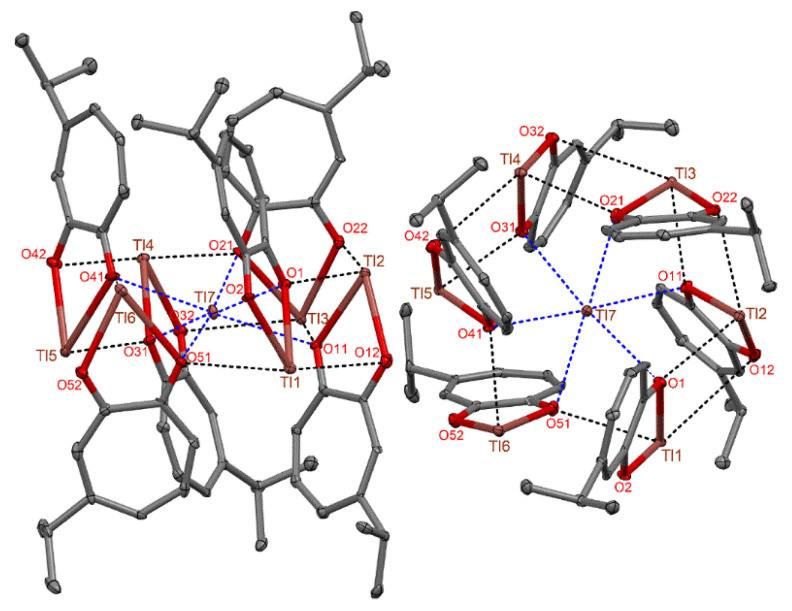
Asymmetric unit of **4** in two different views showing intermolecular contacts between Tl(hino) molecules (black dotted lines) and the shortest distances for the free Tl^+^ ion (blue dotted lines). Atoms are shown as 50% ellipsoids. Hydrogen atoms and the triflate ion were omitted for clarity.

The crystal structure of **3** contained four Tl(hino) molecules in the asymmetric unit (Figure 7), while the asymmetric part of **4** consisted of six such chelate molecules and one thallium(I) cation neutralized by a triflate anion (Figure 8). This Tl^+^ ion (Tl7) was isolated from the CF_3_SO_3_^−^ ion by six Tl(hino) molecules, which formed a specific cage for the free metal cation.

**Figure 9:**
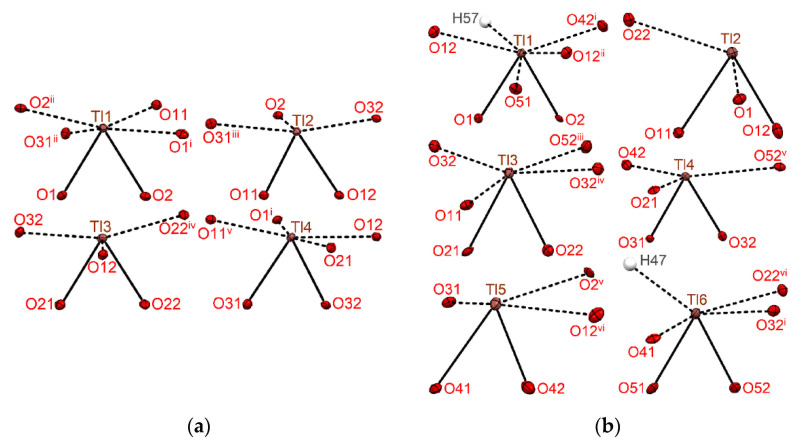
Coordination spheres of thallium(I) ions in compounds (**a**) **3** (symmetry codes as in Table 3) and (**b**) **4** (symmetry codes as in Table 4). Solid and dashed lines indicate chelating bonds and intermolecular contacts, respectively.

Each chelated thallium(I) ion in both compounds was surrounded in its closest environment by five or six oxygen atoms (Figure 9). Two of them belonged to the hinokitiolato ligand, which formed a five-membered chelate ring. There were also three or four additional oxygen atoms from neighboring hino^−^ ligands, while two such oxygen atoms could be distinguished only for Tl2 in **4**. For the chelating Tl–O interactions, a broad bond distance distribution (ranges: 2.464–2.669 Å for **3** and 2.472–2.662 Å for **4**) was observed in both presented compounds (Table 3 and Table 4). The mean Tl–O_(hino)_ bond lengths for the respective chelates in **3** were equal to 2.476(3), 2.571(3), 2.591(3), and 2.608(3) Å for the Tl3, Tl2, Tl1, and Tl4 atoms, respectively. In turn, such mean Tl–O_(hino)_ distances, 2.520(5), 2.504(6), 2.572(5), 2.505(5), 2.578(6), and 2.552(5) Å for the atoms from Tl1 to Tl6, respectively, could be distinguished for complexes in **4**.

In the presented compounds with a hinokitiol ligand, some part of the thallium(I) ions, similarly to **2**, remained clearly outside the plane of the chelate ring, with a displacement value of approximately 0.59 and 0.64 Å for the Tl4 and Tl3 atoms in **3** and 0.27 and 0.54 Å for the Tl5 and Tl3 atoms in **4**.

All Tl(hino) complexes in the extended crystal structures were stabilized by weaker thallium–oxygen contacts between neighboring molecules. These additional interactions are listed in Table 3 and Table 4. Most of such bridging Tl···O distances in **3** and **4** remained in the range of 2.72–3.08 Å, much shorter than the 3.48 Å calculated for the sum of the van der Waals radii of both atoms [37]. A few longer contacts of approximately 3.41, 3.43, and 3.45 Å could also be found in **4**. In addition, some intermolecular anagostic Tl···H–C interactions of about 2.92 (Tl6···H47), 2.93 (Tl1···H57), and 2.99 Å (Tl5···H37), shorter than the accepted sum of the van der Waals radii of Tl and H atoms (3.06 Å [37]), appeared to contribute to the stability of the crystal packing in **4**. The corresponding Tl–H–C angles associated with these contacts were 123 (for Tl6), 133 (Tl1), and 122° (Tl5). These anagostic Tl···H–C interactions found in **4** were shorter than those for other thallium(I) systems presented recently [53,54].

**Figure 10:**
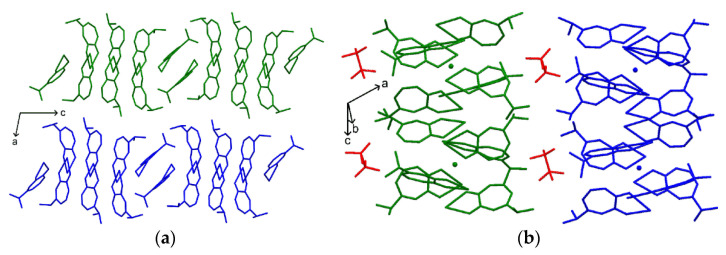
The arrangement of molecules in the extended structures of (**a**) **3** and (**b**) **4**.

The position of the free Tl^+^ ion in **4** was stabilized by six thallium–oxygen contacts with a mean Tl···O distance of 2.906(6) Å and a narrow distance distribution (range: 2.868–2.979(6) Å) showing a distorted octahedral configuration around this metal center. In the crystal structures of both compounds, the Tl(hino) molecules were arranged into layers (Figure 10) with isopropyl groups directed outside of them. In addition, the triflate anions were placed between these layers in **4**.

The shortest Tl···Tl distances between neighboring Tl(hino) molecules in **3** were equal to 3.904(1) Å (Tl1···Tl4), 3.905(1) (Tl2···Tl3), and 4.001(1) Å (Tl3···Tl4), close to double the van der Waals radius of thallium (3.92 Å [37]), and they could be considered as a weak metallophilic bonding. Similar contacts in **4** were a bit longer, in the range of 4.10–4.23 Å, and were formed between the free thallium(I) ion and other metal atoms belonging to the adjacent Tl(hino) chelates. The closest Tl···Tl distances between neighboring complex molecules in **4** were even longer, above 4.25 Å.

Several hinokitiolato–metal complexes have been structurally characterized and tested against antimicrobial activities [7,55].

### 2.3. Spectroscopic Analysis

Comparison of the IR spectrum of the Tl(OTf)·Htrop adduct with the spectra of pure tropolone and thallium(I) triflate components (Figures S1–S3) allowed us to select two groups of the IR bands corresponding to (i) tropolone and to (ii) triflate anion vibrations. The former dominated the spectra of the described system. The broad band at 3170 cm^−1^ could be assigned to the tropolone OH stretching vibrations. The bending and torsional OH modes could be distinguished at 1254 and 759 cm^−1^ [56]. The asymmetric and symmetric stretching tropolone C=O bands could be found at 1607 and 1532 cm^−1^. The main IR bands of the triflate ion were those of the SO_3_ and CF_3_ group vibrations located at 1218, 1154, and 1021 cm^−1^.

The juxtaposition of the IR spectra of tropolone ligands with the reciprocal thallium(I) complexes (Figures S3–S9) showed that the tropolonate bands dominated the spectra. The thallium(I) chelates were formed through the tropolonate C=O and O–H moieties, which were corroborated by the absence of the stretching and bending OH bands at 3187 and 1264, 3190 and 1261, or 3203 and 1268 cm^−1^ in free tropolone, 5-methyltropolone, or hinokitiol, respectively. This was accompanied by a decrease in the double bond character of the C=O group coordinated to the metal ion, manifested by a downshift of the C=O bands at 1603 and 1543 (Htrop) or 1619 and 1542 cm^−1^ (5-meHtrop), respectively, to 1588 and 1492 cm^−1^ or 1605 and 1508 cm^−1^ in the thallium(I) complexes. The analogous hinokitiol bands at 1606 and 1541 cm^−1^ shifted to 1582 and 1490 cm^−1^ in the thallium(I) hinokitiolate complexes (**3** and **4**). 

The UV–vis spectra of tropolone ligands and the **1**–**4** thallium(I) complexes in the methanol solutions were similar to each other (Figure S10). Indeed, as the free ligands, **1**–**4** exhibited intense absorptions below 250 nm and two distinct bands in the 300–420 nm region: more intense at 332 nm and weaker at 395 nm. The additional band could be seen between them as a shoulder at *ca*. 370 nm. In this very region, the bands of complexes were redshifted and more distinct than those of the pure ligands. 

The photophysical data of thallium(I) chelates studied in methanol and the solid state are summarized in Table 5, while the emission and absorption profiles can be found in Figure S11. The fluorescence spectra of all compounds in methanol solution showed very broad profiles, from around 380 up to 550 nm (left panels of Figure S11). In the solid state, two bands around 420 and 462 nm (emission spectra) and 375 and 420 nm (excitation spectra) were observed (right panels of Figure S11). Only **2** exhibited fluorescence that was redshifted (502 nm) in comparison to the other thallium(I) chelates. The fluorescence decays had two components in the solid state: a long-lived one, with a lifetime around 15 ns; and the other short-lived, with a lifetime around 0.15 ns. In the solid-state measurements, the highest average lifetime was found for **4,** and the shortest for **1**. Interestingly, in the methanol solution, this trend was reversed. In methanol, the fluorescence quantum yields φ of the thallium(I) chelates were relatively high. Indeed, the complexes with tropolone and 5-methyltropolone exhibited 3 or 4 times higher quantum yields than for complexes with hinokitiol. Furthermore, all the studied complexes displayed very short-lived emissions τ if compared to, e.g., thallium(I) acetyloacetonate [57]. The photophysical data of the two hinokitiol complexes (**3** and **4**) were almost the same. 

The NMR data of all compounds in solution are gathered in Figures S12–S17. The ^1^H NMR spectra of thallium(I) compounds showed three (Tl(OTf)·Htrop and **1**), five (**2**), or six (**3** and **4**) proton signals originating from the respective ligand. In the case of the adduct, the signal of the OH proton in Htrop was not registered. However, for the [Bi(trop)_2_(Htrop)(CF_3_SO_3_)] and [Pb(trop)_2_(Htrop)] complexes, a broad proton signal of the tropolone OH group was previously observed by us at around 10 ppm [16]. In the ^205^Tl NMR spectra, the Tl^+^ signal from the thallium(I) triflate and its adduct with tropolone was located at 370 ppm (Figure S18). Complexation by tropolonates broadened the signal and shifted it to the lower field by over 1000 ppm. For **4**, **1**, **3**, and **2**, the ^205^Tl NMR signals were observed at 1373, 1416, 1507, and 1574 ppm, respectively (Figure S18). Surprisingly, the ^205^Tl NMR signals of the Tl(hino) chelates **3** and **4** differed by about 130 ppm. Due to the relatively low concentration of the uncoordinated thallium(I) ion in **4,** its signal could not be registered.

### 2.4. Antimicrobial Activity

The in vitro antimicrobial activity of thallium(I) tropolonate complexes and free tropolone ligands was compared with the selected lead(II) and bismuth(III) analogues characterized chemically earlier [14,15,17]. The activity of three thallium(I) (Tl(trop), Tl(5-metrop) and Tl(hino)), two lead(II) (Pb(hino)_2_ and Pb(trop)_2_), and three bismuth(III) ([Bi_2_(trop)_4_(NO_3_)_2_]_n_, Bi(trop)_3_ and Bi(5-metrop)_2_(NO_3_)) complexes and their ligands against two groups of reference microorganisms were examined. The first one was composed of strains recommended by the European Committee on Antimicrobial Susceptibility Testing (EUCAST) for the antibiotics quality control [58]. This group contained microorganisms representative of the most frequent infections in humans: *Escherichia coli* ATCC 25922, *Pseudomonas aeruginosa* ATCC 27853, *Staphylococcus aureus* ATCC 29213, and *Enterobacter faecalis* ATCC 29212. The second group consisted of microorganisms indicated by the United States Pharmacopeia to test the antimicrobial effectiveness [59]. It contained the following strains: *E. coli* ATCC 8739, *P. aeruginosa* ATCC 9027, *S. aureus* ATCC 6538, and *Candida albicans* ATCC 10231. The minimum inhibitory concentration (MIC) values for the two groups of bacterial species were compared with the results included in EUCAST breakpoint tables for interpretation of MICs (Table 6).

Surprisingly, none of the thallium(I) complexes displayed potent antimicrobial activity. Moreover, the free 5-meHtrop ligand had high activity against *E. faecalis* ATCC 29212 (MIC = 2 mg·mL^−1^), whereas the activity of the Tl(5-metrop) complex was significantly reduced (MIC = 256 mg·mL^−1^). This suggested that the α-hydroxycarbonyl moiety with neighboring OH and C=O groups, present in the free ligand and blocked in the thallium(I) complex, played a crucial role in the antimicrobial activity of the free 5-meHtrop ligand. In addition, the two lead(II) complexes showed no significant activity against the tested microorganisms. In contrast, but in line with previous reports [60,61,62], the bismuth(III) complexes demonstrated significant antimicrobial activity. It was found earlier that some bismuth(III) tropolonates showed higher activity against *Helicobacter pylori* in the agar dilution test than the standard bismuth(III) salicylate [13]. Nevertheless, the bismuth(III) hinokitiolate complex did not demonstrate a specific antimicrobial action [7]. The mechanism of the bismuth compounds antimicrobial activity is not fully understood. Yet, it is known that they increase the permeability of the bacterial cell membrane and reduce the glycocalyx production [63,64]. All tested bismuth(III) complexes showed 2- to 5-fold larger activity than their free ligands (Table 6). In the case of *S. aureus* ATCC 29213, the MIC values were 8 mg·mL^−1^ for the bismuth(III) complexes, and 128 mg·mL^−1^ for ligands. For this very reference strain, the MIC cut-off values of the majority of the antibiotics administrated for treatment were significantly lower. However, for chloramphenicol, an antibiotic used to treat staphylococci infections, the breakpoint was 8 mg·mL^−1^ as well. The same MIC value was also demonstrated by the Bi(trop)_3_ complex against *E. coli* ATCC 25922 and *E. faecalis* ATCC 29212, and also by the Bi(5-metrop)_2_(NO_3_) complex against *S. aureus* ATCC 6538, *E. coli* ATCC 25922, and *P. aeruginosa* ATCC 9027. On the other hand, for amikacin, a broad-spectrum antibiotic to treat infections caused by Gram-negative and Gram-positive bacteria, the breakpoint against *P. aeruginosa* was 16 mg·mL^−1^. Thus, the Bi(5-metrop)_2_(NO_3_) complex displayed a two-times lower MIC.

Much less satisfactory were the minimum bactericidal concentration values; MBC = 16 mg·mL^−1^ was obtained for [Bi_2_(trop)_4_(NO_3_)_2_]_n_ and Bi(trop)_3_ complexes against *E. coli* ATCC 25922, *S. aureus* ATCC 29213, and *S. aureus* ATCC 6538. Moreover, the two complexes exhibited MBC = 64 mg·mL^−1^ against *P. aeruginosa* ATCC 27853 and *P. aeruginosa* ATCC 9027. The Bi(trop)_3_ complex showed a killing activity of 256 mg·mL^−1^ with MBC for *E. coli* ATCC 8739. However, the MBC for the Tl(hino) complex was equal to 512 mg·mL^−1^ against both: *E. coli* strains, ATCC 25922, ATCC 8739, and *E. faecalis* ATCC 29212, but the other thallium(I) complexes did not show a killing effect in the tested range of concentrations (Table 6).

## 3. Materials and Methods

**Scheme 1:**
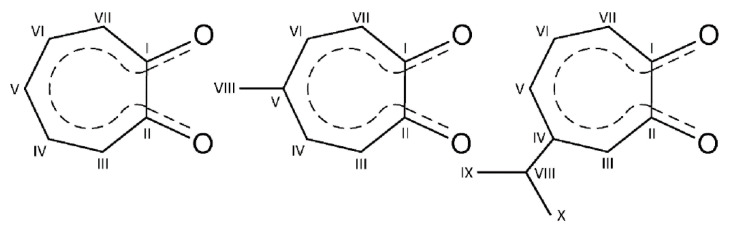
Atom labeling in tropolonato-like ligands used for NMR assignments.

The 5-Methyltropolone was synthesized as presented earlier [52]. All the other reagents were used as purchased from commercial sources. The IR spectra were recorded on a Thermo Scientific Nicolet iS10 FT-IR spectrometer (Thermo Fisher Scientific, Waltham, MA, USA) in the 4000–650 cm^−1^ range with 4 cm^−1^ resolution using a PIKE Technologies MIRacle (Madison, WI, USA) accessory equipped with ZnSe crystal designed for single reflection horizontal ATR technique. UV–vis spectra were registered with Thermo Scientific Evolution 600 (Thermo Fisher Scientific, Waltham, MA, USA) or Perkin-Elmer Lambda 35 spectrometers (Waltham, MA, USA) using methanol solutions of the studied complexes and ligands. Emission spectra and fluorescence lifetimes at room temperature were collected on a Horiba Fluorolog 3 fluorimeter (Horiba Scientific, Piscataway, NJ, USA). Excitation for fluorescence lifetime measurements was provided by Delta Diode 336 and 303 nm for solutions and solid states, respectively. The ^1^H, ^13^C, ^19^F, and ^205^Tl NMR spectra of DMSO-*d*_6_ solutions were recorded at 298 K on a Bruker AVANCE III HD 500 MHz spectrometer (Bruker, Billerica, MA, USA). An aqueous solution of thallium(I) nitrate (1 mol·dm^−3^) was used as a chemical shift reference (0 ppm). The numbering of carbon atoms in the description of NMR signals is shown in Scheme 1.

### 3.1. Preparation of the Thallium(I) Compounds

#### 3.1.1. Tl(CF_3_SO_3_) (Tl(OTf))

The thallium(I) triflate was synthesized in the reaction of metallic thallium with concentrated aqueous solution of trifluoromethanesulfonic acid, followed by evaporation of the excess of water and acid. ATR-IR (ZnSe), ν_max_/cm^−1^: 1217 vs (SO_3_), 1160 m (CF_3_), 1015 m (SO_3_), 763 w (CF_3_); assignments based on [65]. ^13^C NMR (125.79 MHz, DMSO-*d_6_*) *δ*/ppm: 120.70 (CF_3_). ^19^F NMR (470.63 MHz, DMSO-*d_6_*) *δ*/ppm: −77.73 (CF_3_). 

#### 3.1.2. Co-Crystals of Thallium(I) Triflate and Tropolone-Tl(OTf)·Htrop

The titled crystals were obtained from the methanol solution (0.3 mL) containing thallium(I) triflate (0.151 g, 0.427 mmol) and tropolone (0.052 g, 0.426 mmol) at a molar ratio of 1:1. The prepared solution was stored in a fridge until almost all of the solvent was evaporated off, giving colorless, platelet-shaped crystals. Yield: 0.190 g, 94.1% (based on Tl). Anal. *Calc.* for C_8_H_6_F_3_O_5_STl: C, 20.20; H, 1.27; S, 6.74. Found: C, 20.21; H, 1.29; S, 6.78%. ATR-IR (ZnSe), ν_max_/cm^−1^: 3170 br, 3015 br, 1607 m, 1559 vw, 1532 w, 1484 m, 1458 sh, 1433 m, 1307 vw, 1254 sh, 1233 s, 1218 vs, 1154 vs, 1056 vw, 1021 m, 960 w, 932 vw, 878 w, 768 w, 759 sh, 716 w, 688 w, 669 vw. ^1^H NMR (500.20 MHz, DMSO-*d_6_*) *δ*/ppm: 7.05 (t, 1H, H^V^), 7.22 (dd, 2H, H^III,VII^), 7.41 (ddd, 2H, H^IV,VI^). ^13^C NMR (125.79 MHz, DMSO-*d_6_*) *δ*/ppm: 124.42 (C^III,VII^), 127.95 (C^IV,VI^), 137.32 (C^V^), 171.80 (C^I,II^). ^19^F NMR (470.63 MHz, DMSO-*d_6_*) *δ*/ppm: −77.73 (CF_3_).

#### 3.1.3. Tl(trop) (**1**)

Thallium(I) nitrate (0.100 g, 0.375 mmol) was dissolved in hot water (1.0 mL), and to this solution, tropolone (0.045 g, 0.377 mmol) in methanol (0.5 mL) was added. To the resulting mixture, an aqueous ammonia solution (28 µL, 25%, 0.376 mmol NH_3_) dissolved in methanol (0.2 mL) was slowly added dropwise. The whole mixture was stirred at all times. During the addition of ammonia solution, the mixture had already turned yellow, and a yellow crystalline material of **1** began to precipitate. After a few hours, this compound was isolated from the solution. Yield: 0.058 g, 47.5% (based on Tl). Anal. *Calc.* for C_7_H_5_O_2_Tl: C, 25.83; H, 1.55. Found: C, 25.78; H, 1.68%. UV–vis (methanol), *λ*_max_/nm (*ε*/M^−1^·cm^−1^): 332 (12 800), 370*sh* (6000), 396 (6050). ATR-IR (ZnSe), ν_max_/cm^−1^: 3029 vw, 3008 vw, 2986 vw, 1588 m, 1492 m, 1466 sh, 1429 sh, 1412 sh, 1389 s, 1380 sh, 1358 m, 1348 sh, 1229 sh, 1217 vs, 995 vw, 970 w, 898 vw, 877 w, 728 m, 721 sh, 679 vw. ^1^H NMR (500.20 MHz, DMSO-*d_6_*) *δ*/ppm: 6.54 (dd, 1H, H^V^), 6.77 (d, 2H, H^III,VII^), 7.62 (dd, 2H, H^IV,VI^). ^13^C NMR (125.79 MHz, DMSO-*d_6_*) *δ*/ppm: 120.30 (C^V^), 124.81 (C^III,VII^), 135.32 (C^IV,VI^), 183.10 (C^I,II^).

#### 3.1.4. Tl(5-metrop) (**2**)

Thallium(I) triflate (0.039 g, 0.110 mmol) and 5-methyltropolone (0.015 g, 0.110 mmol) were dissolved in methanol (1.0 mL). Then, a solution of ammonia (8.3 µL, 25% in H_2_O) in methanol (0.2 mL) was slowly added to the stirred mixture. During the addition of the ammonia solution, lemon-yellow crystals were precipitated. Yield: 0.012 g, 32.4% (based on Tl). Anal. *Calc.* for C_8_H_7_O_2_Tl: C, 28.30; H, 2.08. Found: C, 28.30; H, 2.10%. UV–vis (methanol), *λ*_max_/nm (*ε*/M^−1^·cm^−1^): 332 (8330), 372*sh* (4150), 401 (2100). ATR-IR (ZnSe), ν_max_/cm^−1^: 3015 vw, 2915 vw, 1605 w, 1594 sh, 1546 vw, 1508 m, 1496 sh, 1453 sh, 1425 s, 1396 sh, 1379 w, 1363 m, 1338 vs, 1238 m, 1220 sh, 1124 w, 1041 vw, 992 vw, 957 vw, 895 vw, 872 vw, 849 vw, 824 m, 765 w, 738 m, 668 vw. ^1^H NMR (500.20 MHz, DMSO-*d_6_*) *δ*/ppm: 2.28 (s, 3H, H^VIII^), 6.75 (d, 2H, H^III,VII^), 7.10 (d, 2H, H^IV,VI^). ^13^C NMR (125.79 MHz, DMSO-*d_6_*) *δ*/ppm: 24.31 (C^VIII^), 125.44 (C^III,VII^), 129.68 (C^V^), 136.34 (C^IV,VI^), 182.14 (C^I,II^).

#### 3.1.5. Tl(hino) (**3**)

Thallium(I) nitrate (0.050 g, 0.188 mmol) was dissolved in hot water (0.4 mL) and mixed with a solution of hinokitiol (0.031 g, 0.189 mmol) in methanol (1.0 mL). Then, an ammonia solution (29 µL, 25% in H_2_O, ~0.188 mmol) dissolved in methanol (0.1 mL) was slowly added, and the whole mixture was stirred and slightly heated for about 2 h. After the addition of ammonia, a yellow compound began to precipitate. Then, the solution was stored at room temperature, and light-yellow needlelike crystals of **3** were separated after a few days. Yield: 0.054 g, 78.3% (based on Tl). Anal. *Calc.* for C_10_H_11_O_2_Tl: C, 32.68; H, 3.02. Found: C, 32.52; H, 2.87%. UV–vis (methanol), *λ*_max_/nm (*ε*/M^−1^·cm^−1^): 332 (9600), 365*sh* (5450), 391 (3500). ATR-IR (ZnSe), ν_max_/cm^−1^: 3025 vw, br, 2958 w, 2929 vw, br, 2885 vw, 2865 vw, 1581 m, 1489 m, 1450 m, 1416 sh, 1392 s, 1381 sh, 1358 s, 1318 vw, 1308 sh, 1291 m, 1230 vs, 1187 vw, 1125 vw, 1102 vw, 1189 vw, 1064 vw, 1045 vw, 1015 vw, 957 w, 905 vw, 895 vw, 889 vw, 876 sh, 807 w, 768 w, 751 vw, 747 vw, sh, 721 w, 669 vw. ^1^H NMR (500.20 MHz, DMSO-*d_6_*) *δ*/ppm: 1.18 (d, 6H, H^IX,X,^), 2.74 (m, 1H, H^VIII^), 6.52 (dd, 1H, H^V^), 6.66 (d, 1H, H^VII^), 6.78 (d, 1H, H^III^), 7.12 (dd, 1H, H^VI^). ^13^C NMR (125.79 MHz, DMSO-*d_6_*) *δ*/ppm: 23.78 (C^IX,X^), 38.20 (C^VIII^), 119.96 (C^III^), 123.49 (C^V^), 124.63 (C^VI^), 135.12 (C^VII^), 156.14 (C^IV^), 181.98 (C^I^), 186.66 (C^II^).

#### 3.1.6. Tl@[Tl(hino)]_6_}(OTf) (**4**)

The titled compound was obtained from methanol solution (1.0 mL) in a similar way as for **3** using the following amounts of reagents: thallium(I) triflate (0.050 g, 0.141 mmol), hinokitiol (0.023 g, 0.140 mmol), and ammonia (21 µL, 25% in H_2_O, ~0.136 mmol). The volume of the reaction mixture was reduced under warming to about half of its starting volume. Yellow platelike crystals were separated from this solution after storing it for a few days at room temperature. Yield: 0.018 g, 34.6% (based on Tl). Anal. *Calc.* for C_61_H_66_F_3_O_15_STl_7_: C, 28.63; H, 2.60; S, 1.25. Found: C, 28.67; H, 2.56; S, 1.27%. UV–vis (methanol), *λ*_max_/nm (*ε*/M^−1^·cm^−1^, based on the concentration of Tl(hino)): 332 (8571), 365*sh* (4151), 391 (4087). ATR-IR (ZnSe), ν_max_/cm^−1^: 2964 vw, br, 2878 vw, 1584 w, 1574 sh, 1559 sh, 1494 m, 1455 sh, 1446 m, 1438 sh, 1404 s, 1383 m, 1357 m, 1337 m, 1315 w, 1275 s, 1238 vs, 1185 vw, 1143 m, 1109 vw, 1092 vw, 1033 w, 965 w, 908 vw, 889 vw, 814 w, 766 w, 755 w, 721 sh, 713 w, 669 vw. ^1^H NMR (500.20 MHz, DMSO-*d_6_*) *δ*/ppm: 1.18 (d, 6H, H^IX,X^), 2.74 (m, 1H, H^VIII^), 6.52 (dd, 1H, H^V^), 6.65 (d, 1H, H^VII^), 6.78 (d, 1H, H^III^), 7.12 (dd, 1H, H^VI^). ^13^C NMR (125.79 MHz, DMSO-*d_6_*) *δ*/ppm: 23.78 (C^IX,X^), 38.20 (C^VIII^), 119.91 (C^III^), 123.51 (C^V^), 124.65 (C^VI^), 135.11 (C^VII^), 156.13 (C^IV^), 182.10 (C^I^), 186.66 (C^II^). ^19^F NMR (470.63 MHz, DMSO-*d_6_*) *δ*/ppm: −77.74 (CF_3_).

### 3.2. Single-Crystal X-ray Diffraction

X-ray diffraction data for the obtained crystals were collected at 100 K on a Rigaku SuperNova (dual source) diffractometer equipped with an Eos CCD detector using the mirror-monochromated Mo Kα or Cu Kα radiations (λ = 0.71073 or 1.54184 Å) from a microfocus Mova or Nova X-ray sources, respectively. A suitable single crystal was mounted on a nylon loop using cryoprotectant oil (Paratone-N). The majority of the solid Tl(OTf) compound was noncrystalline, giving no reflections in the course of pre-experiment during the X-ray analysis. However, it succeeded in finding the more ordered material suitable for scXRD measurement. Crystallographic parameters and refinement details for all studied compounds are given in Table S1. Data collection and reduction were performed using CrysAlis PRO software. For the crystals, the empirical absorption correction using spherical harmonics, implemented in the SCALE3 ABSPACK scaling algorithm, was introduced. The crystal structures were solved by the direct method and refined by the full-matrix least-squares method on *F*^2^ data. All nonhydrogen atoms (except for O3, O4, O5, O6, and C1 atoms in Tl(OTf)) were refined with anisotropic atomic displacement parameters. All H atoms bonded to C atoms were placed in calculated positions with C–H = 0.95 (aromatic), 0.98 (methyl), or 1.00 Å (methine) and refined isotropically using a riding model with *U*_iso_(H) = 1.5 *U*_eq_(C) for the methyl H atoms and 1.2 *U*_eq_(C) for the remaining H atoms. The H atom of the OH group in Tl(OTf)·Htrop was located in a difference map, and its position and isotropic thermal parameter *U*_iso_ were freely refined. All calculations were performed with the SHELXTL program package [66] integrated with the OLEX2 crystallographic software [67]. The MERCURY program [68] was applied for the structural graphics.

### 3.3. Antimicrobial Activity

The in vitro antimicrobial activity of ligands and complexes dissolved in DMSO was determined according to ISO 20776-1 [69]. The minimum inhibitory concentrations (MICs) and minimum bactericidal/fungicidal concentrations (MBCs/MFCs) were tested for reference microorganisms obtained from the ATCC Culture Collection [70]: *E. coli* ATCC 8739, *E. coli* ATCC 25922, *P. aeruginosa* ATCC 9027, *P. aeruginosa* ATCC 27853, *S. aureus* ATCC 6538, *S. aureus* ATCC 29213, *E. faecalis* ATCC 29212, and *C. albicans* ATCC 10232. The procedure consisted of the preparation of 2-fold dilutions of the tested compounds in liquid growth medium dispensed into a 96-well microtiter plate. Serial dilutions were carried out from 512 to 0.5 mg∙mL^−1^ and inoculated with standardized microbial suspension (adjusted to 0.5 McFarland scale) prepared in the same media in a total volume of 200 mL per well. Culture plates were incubated at 35 °C for 18 h. The lowest concentration of compound inhibiting visible bacterial/fungal growth was referred to as the MIC, and that causing inoculum death was reported as the MBC/MFC.

## 4. Conclusions

Thallium(I) triflate (Tl(OTf)) was synthesized in the reaction of the concentrated aqueous solution of trifluoromethanesulfonic acid with metallic thallium. Co-crystals of thallium(I) triflate and tropolone (Tl(OTf)·Htrop) were obtained from a mixture of both compounds at a 1:1 molar ratio. Reactions of tropolone (Htrop) and 5-methyltropolone (5-meHtrop) with thallium(I) triflate or nitrate(V) in methanol solution after addition of an equimolar amount of ammonia led to the formation of the simple chelates: Tl(trop) (**1**) and Tl(5-metrop) (**2**). In the case of 4-isopropyltropolone (hinokitiol, Hhino), a similar process gave the simple Tl(hino) complex (**3**) when using thallium(I) nitrate(V) and the more complex compound {Tl@[Tl(hino)]_6_}(OTf) (**4**) for the thallium(I) triflate salt. The crystal structures of thallium(I) triflate, its co-crystal with tropolone, and complexes **1**–**4** were determined by means of single-crystal X-ray diffraction and characterized by FT-IR, NMR (^1^H, ^13^C, ^19^F, and ^205^Tl), UV–vis, and luminescence techniques. Antimicrobial activity of thallium(I) complexes was tested and compared with free ligands and selected analogous lead(II) and bismuth(III) compounds. None of the thallium(I) complexes showed significant antimicrobial activity. Of the other compounds tested, only the bismuth(III) complexes presented potentially interesting activity, two to five times larger than their free ligands.

## Figures and Tables

**Table 1:** Selected bond lengths, distances (Å), and angles (°) for thallium(I) triflate and its adduct with tropolone.

Tl(OTf)			
Tl1···O1	2.784(18)	Tl2···O6	2.782(16)
Tl1···O4	2.721(14)	Tl2···O2^i^	2.798(16)
Tl1···O1^i^	2.89(2)	Tl2···O4^iii^	3.06(2)
Tl1···O2^i^	3.09(2)	Tl2···O5^iii^	2.91(2)
Tl1···O3^ii^	2.98(2)	Tl2···O3^v^	2.85(1)
Tl1···O5^iii^	2.85(2)	Tl2···O6^vi^	2.93(2)
Tl1···F3^ii^	3.36(1)	Tl2···F3^iii^	3.22(1)
Tl1···F4^iv^	3.43(1)	Tl2···F5^iv^	3.35(1)
Symmetry codes: (i) −*x*, −*y* + 1, −*z* + 2; (ii) –*x* − 1, −*y* + 1, −*z* + 2; (iii) −*x*, −*y* + 1, −*z* + 1; (iv) *x, y +* 1*, z*; (v) *x* + 1, *y*, *z* − 1; (vi) −*x +* 1, −*y* + 1, −*z* + 1			
**Tl(OTf)·Htrop**			
Tl1···O1	2.722(2)	C1–O1	1.271(4)
Tl1···O3	2.942(2)	C2–O2	1.348(4)
Tl1···O2^i^	3.041(2)	O1–Tl1–O5^ii^	135.53(7)
Tl1···O5^ii^	2.934(2)	O3-Tl1–O1^iii^	147.97(7)
Tl1···O1^iii^	2.744(2)	O2^i^–Tl1–O4^iv^	155.37(6)
Tl1···O1^iv^	2.871(2)		
(i) −*x* + 1, −*y* + 1, −*z* + 1, (ii) −*x* + 1, −*y* + 1, −*z* + 2, (iii) −*x*, −*y* + 1, −*z* + 1; (iv) *x* − 1, *y*, *z*			

**Table 2:** Selected bond lengths, distances (Å), and angles (°) for thallium(I) chelates **1** and **2**.

Bond Lengths		Distances		Angles	
**1**					
Tl1–O1	2.588(3)	Tl1···O1^ii,iv^	2.906(3)	O1–Tl1–O1^i^	61.12(13)
Tl1–O1^i^	2.588(3)	Tl1···O1^iii,v^	2.947(3)		
C1–O1	1.283(5)				
Symmetry codes: (i) −*x* + 1, *y*, −*z* + 1.5; (ii) *x*, −*y +* 2, *z* + 0.5; (iii) −*x* + 1.5, −*y +* 2, *z*; (iv) −*x* + 1, −*y +* 2, −*z* + 1; (v) *x*−0.5, −*y +* 2, −*z* + 1.5					
**2**					
Tl1–O2	2.446(6)	Tl1···O2^i^	2.873(10)	O1–Tl1–O2	65.7(3)
Tl1–O1	2.499(9)	Tl1···O2^ii^	2.888(10)		
C2–O2	1.284(17)	Tl1···O1^iii^	3.189(9)		
C1–O1	1.296(14)				
(i) −*x* + 0.5, *y* − 0.5, −*z* + 1; (ii) −*x* + 0.5, *y* + 0.5, −*z* + 1; (iii) *x*, *y* + 1, *z*					

**Table 3:** Selected bond lengths, distances (Å), and angles (°) for compound **3**.

Bond Lengths			
Tl1–O1	2.616(3)	C1–O1	1.261(5)
Tl1–O2	2.566(3)	C2–O2	1.287(6)
Tl2–O11	2.538(3)	C11–O11	1.266(6)
Tl2–O12	2.604(3)	C12–O12	1.269(5)
Tl3–O21	2.464(3)	C21–O21	1.278(6)
Tl3–O22	2.487(3)	C22–O22	1.266(6)
Tl4–O31	2.669(4)	C31–O31	1.268(6)
Tl4–O32	2.547(3)	C32–O32	1.274(5)
**Distances**			
Tl1···O11	2.867(3)	Tl3···O12	2.755(3)
Tl1···O1^i^	2.930(4)	Tl3···O32	2.986(3)
Tl1···O2^ii^	3.011(3)	Tl3···O22^iv^	3.322(3)
Tl1···O31^ii^	3.044(3)	Tl4···O21	2.726(3)
Tl2···O2	2.719(3)	Tl4···O11^v^	2.971(3)
Tl2···O32	2.803(3)	Tl4···O12	2.971(3)
Tl2···O31^iii^	2.989(4)	Tl4···O1^i^	3.030(3)
**Angles**			
O1–Tl1–O2	61.0(1)	O21–Tl3–O22	64.5(1)
O11–Tl2–O12	61.4(1)	O31–Tl4–O32	60.2(1)

Symmetry codes: (i) −*x* + 0.5, *y*−0.5, −*z* + 0.5; (ii) −*x* + 0.5, *y* + 0.5, −*z* + 0.5; (iii) *x*, *y* + 1, *z*; (iv) −*x* + 0.5, −*y +* 1.5, −*z* + 1; (v) *x*, *y*−1, *z.*

**Table 4:** Selected bond lengths, distances (Å), and angles (°) for compound **4**.

Bond Lengths				Distances			
Tl1–O1	2.567(5)	C1–O1	1.297(9)	Tl1···O42^i^	2.898(6)	Tl5···O31	2.712(5)
Tl1–O2	2.472(5)	C2–O2	1.259(8)	Tl1···O12^ii^	2.905(6)	Tl5···O12^vi^	3.002(6)
Tl2–O11	2.532(5)	C11–O11	1.262(9)	Tl1···O51	3.016(6)	Tl5···O2^v^	3.017(6)
Tl2–O12	2.477(6)	C12–O12	1.280(9)	Tl1···O12	3.446(6)	Tl6···O41	2.831(6)
Tl3–O21	2.652(5)	C21–O21	1.303(9)	Tl2···O1	2.772(7)	Tl6···O32^i^	2.960(6)
Tl3–O22	2.492(5)	C22–O22	1.284(9)	Tl2···O22	2.947(6)	Tl6···O22^vi^	3.019(6)
Tl4–O31	2.522(5)	C31–O31	1.293(9)	Tl3···O11	2.836(6)	Tl7···O41	2.868(6)
Tl4–O32	2.488(5)	C32–O32	1.273(8)	Tl3···O52^iii^	2.867(6)	Tl7···O1	2.878(6)
Tl5–O41	2.662(5)	C41–O41	1.271(9)	Tl3···O32^iv^	2.985(6)	Tl7···O51	2.897(6)
Tl5–O42	2.495(6)	C42–O42	1.287(9)	Tl3···O32	3.430(6)	Tl7···O21	2.906(6)
Tl6–O51	2.621(5)	C51–O51	1.265(9)	Tl4···O21	2.822(6)	Tl7···O31	2.911(6)
Tl6–O52	2.483(5)	C52–O52	1.279(9)	Tl4···O42	3.075(6)	Tl7···O11	2.979(6)
**Angles**				Tl4···O52^v^	3.410(5)		
O1–Tl1–O2	63.9(2)	O31–Tl4–O32	63.8(2)				
O11–Tl2–O12	63.1(2)	O41–Tl5–O42	61.0(2)				
O21–Tl3–O22	61.7(2)	O51–Tl6–O52	62.7(2)				

Symmetry codes: (i) −x + 1, y − 0.5, −z + 0.5; (ii) −x + 1, −y, −z + 1; (iii) x, −y + 0.5, z + 0.5; (iv) −x + 1, −y + 1, −z + 1; (v) −x + 1, y + 0.5, −z + 0.5; (vi) x, −y + 0.5, z − 0.5.

**Table 5:** Photophysical data of thallium(I) chelates.

Compound	Matrix	λ_abs max_/λ_em max_ [nm]	φ [%]	τ [ns]
**1**	MeOH	393/421	0.20 ^a^	0.24
	solid	375,434/411,461	-	8.2 (31%), 0.19 (69%)
**2**	MeOH	402/432	0.27 ^b^	0.22
	solid	375,460/430,502	-	12 (57%), 0.15 (43%)
**3**	MeOH	390/419	0.06 ^b^	0.12
	solid	377,420/417,463	-	16 (62%), 0.12 (38%)
**4**	MeOH	390/421	0.06 ^b^	0.12
	solid	372,426/415,462	-	16 (61%), 0.34 (39%)

^a^ Quinine sulfate in 0.5M H_2_SO_4_; ^b^ perylene in cyclohexane as standards.

**Table 6:** MICs/MBCs * or MFCs * of thallium(I), lead(II), and bismuth(III) complexes and free ligands obtained for reference strains.

	*E. coli* ATCC 8739	E. *coli* ATCC 25922	*S. aureus* ATCC 6538	*S. aureus* ATCC 29213	*P. aeruginosa* ATCC 9027	*P. aeruginosa* ATCC 27853	*E. faecalis* ATCC 29212	*C. albicans* ATCC 10232
Tl(trop)	256/ND	128/ND	256/ND	512/ND	256/ND	128/ND	512/ND	128/ND
Pb(trop)_2_	128/ND	64/ND	128/ND	128/ND	512/ND	256/ND	64/ND	64/ND
Bi(trop)_3_	16/256	8/16	16/16	8/16	32/64	16/64	8/ND	512/ND
[Bi_2_(trop)_4_(NO_3_)_2_]_n_	16/ND	16/16	16/16	8/16	32/64	32/64	16/ND	128/ND
Htrop	128/ND	64/ND	256/ND	128/ND	256/ND	256/ND	256/256	64/512
Tl(5-metrop)	128/ND	64/ND	256/ND	256/ND	128/ND	128/ND	256/ND	64/ND
Bi(5-metrop)_2_(NO_3_)	16/ND	8/ND	8/ND	8/ND	8/ND	16/ND	2/ND	32/ND
5-meHtrop	32/512	16/ND	128/ND	128/ND	128/ND	256/ND	2/256	32/512
Tl(hino)	128/512	128/512	128/ND	64/ND	256/ND	256/ND	64/512	32/ND
Pb(hino)_2_	64/ND	64/ND	128/ND	256/ND	512/ND	512/ND	64/ND	64/ND
Hhino	64/256	32/128	64/256	128/ND	256/ND	256/ND	32/ND	32/512

* ND: killing effect not detected in the tested range of concentrations.

## Data Availability

CCDC 2126510-2126515 contain the supplementary crystallographic data for this paper. These data can be obtained free of charge from the Cambridge Crystallographic Data Center at http://www.ccdc.cam.ac.uk/data_request/cif (accessed on 6 December 2021).

## Supplementary Materials

The following are available online, Table S1: Crystal data and structure refinement details for studied thallium(I) compounds; Figure S1: ATR-FTIR of Tl(OTf); Figure S2: ATR-FTIR of Tl(OTf)∙Htrop adduct; Figure S3: ATR-FTIR of Htrop; Figure S4: ATR-FTIR of Tl(trop) (**1**); Figure S5: ATR-FTIR of 5-meHtrop; Figure S6: ATR-FTIR of Tl(5-metrop) (**2**); Figure S7: ATR-FTIR of Hhino; Figure S8: ATR-FTIR of Tl(hino) (**3**); Figure S9: ATR-FTIR of {Tl@[Tl(hino)]_6_}(OTf) (**4**); Figure S10: Experimental UV–vis absorption spectra of the studied thallium(I) complexes (red lines) in comparison with the respective ligand (black lines) measured in MeOH solutions; Figure S11: Excitation and emission spectra for complexes **2**–**5** in methanol (left) and the solid state (right); Figure S12: ^13^C NMR spectrum of Tl(OTf); Figure S13: (a) ^1^H NMR, (b) ^13^C NMR, and (c) ^19^F NMR spectra of Tl(OTf)∙Htrop adduct; Figure S14: (a) ^1^H NMR and (b) ^13^C NMR spectra of Tl(trop) (**1**); Figure S15: (a) ^1^H NMR and (b) ^13^C NMR spectra of Tl(5-metrop) (**2**); Figure S16: (a) ^1^H NMR and (b) ^13^C NMR spectra of Tl(hino) (**3**); Figure S17: (a) ^1^H NMR, (b) ^13^C NMR, and (c) ^19^F NMR spectra of {Tl@[Tl(hino)]_6_}(OTf) (**4**); Figure S18: ^205^Tl NMR spectra of studied thallium(I) compounds.

## Sample Availability

Samples of the compounds in this article are not available from the authors.
