# Supplementary material for: Thallium(I) Tropolonates: Synthesis, Structure, Spectral Characteristics, and Antimicrobial Activity Compared to Lead(II) and Bismuth(III) Analogues
Source: Molecules. 2021 Dec 29;27(1):183. doi: 10.3390/molecules27010183 (PMC8746424; doi:10.3390/molecules27010183)

checkCIF/PLATON report

Structure factors have been supplied for datablock(s) tl7hino6otf, tlhino, tlmetrop, tlotf, tlotf-htrop, tltrop

THIS REPORT IS FOR GUIDANCE ONLY. IF USED AS PART OF A REVIEW PROCEDURE FOR PUBLICATION, IT SHOULD NOT REPLACE THE EXPERTISE OF AN EXPERIENCED CRYSTALLOGRAPHIC REFEREE.

No syntax errors found. CIF dictionary Interpreting this report

Datablock: tlotf

```
Bond precision:   F- C = 0.0300 A          Wavelength=0.71073

Cell:           a=7.5368(15)        b=8.3722(18)       c=9.3994(19)
                alpha=88.170(17)    beta=73.168(18)    gamma=89.134(17)
Temperature:    100 K

                 Calculated                    Reported
Volume           567.4(2)                      567.4(2)
Space group      P -1                          P -1
Hall group       -P 1                          -P 1
Moiety formula   C2 F6 O6 S2 Tl2               C2 F6 O6 S2 Tl2
Sum formula      C2 F6 O6 S2 Tl2               C2 F6 O6 S2 Tl2
Mr               706.90                        706.88
Dx,g cm-3        4.138                         4.138
Z                2                             2
Mu (mm-1)        28.830                        28.830
F000             616.0                         616.0
F000'            606.53
h,k,lmax         10,12,13                      10,11,13
Nref             3675                          2684
Tmin,Tmax        0.008,0.056                   0.076,1.000
Tmin'            0.004

Correction method= # Reported T Limits: Tmin=0.076 Tmax=1.000
AbsCorr = MULTI-SCAN

Data completeness= 0.730           Theta(max)= 31.208

R(reflections)= 0.0774( 1953)                      wR2(reflections)= 0.2173( 2684)

S = 1.041                  Npar= 138
```

The following ALERTS were generated. Each ALERT has the format
test-name_ALERT_alert-type_alert-level.
Click on the hyperlinks for more details of the test.

Alert level A

PLAT029_ALERT_3_A _diffrn_measured_fraction_theta_full value Low . 0.880 Why?
PLAT971_ALERT_2_A Check Calcd Resid. Dens. 1.05A From Tl1 5.46 eA-3
PLAT971_ALERT_2_A Check Calcd Resid. Dens. 0.89A From Tl2 5.25 eA-3
PLAT971_ALERT_2_A Check Calcd Resid. Dens. 1.16A From Tl2 5.19 eA-3
PLAT971_ALERT_2_A Check Calcd Resid. Dens. 0.93A From Tl1 4.79 eA-3
PLAT971_ALERT_2_A Check Calcd Resid. Dens. 0.76A From Tl2 4.65 eA-3
PLAT971_ALERT_2_A Check Calcd Resid. Dens. 0.93A From Tl2 4.19 eA-3
PLAT971_ALERT_2_A Check Calcd Resid. Dens. 1.00A From Tl1 3.99 eA-3
PLAT971_ALERT_2_A Check Calcd Resid. Dens. 0.76A From Tl2 3.96 eA-3
PLAT971_ALERT_2_A Check Calcd Resid. Dens. 0.77A From Tl1 3.86 eA-3
PLAT971_ALERT_2_A Check Calcd Resid. Dens. 0.88A From Tl1 3.58 eA-3
PLAT972_ALERT_2_A Check Calcd Resid. Dens. 0.73A From Tl2 -5.16 eA-3
PLAT972_ALERT_2_A Check Calcd Resid. Dens. 0.85A From Tl2 -4.40 eA-3
PLAT972_ALERT_2_A Check Calcd Resid. Dens. 0.81A From Tl1 -4.40 eA-3
PLAT972_ALERT_2_A Check Calcd Resid. Dens. 0.60A From Tl1 -4.19 eA-3
PLAT972_ALERT_2_A Check Calcd Resid. Dens. 0.74A From Tl1 -4.09 eA-3
PLAT972_ALERT_2_A Check Calcd Resid. Dens. 0.88A From Tl2 -4.01 eA-3
PLAT972_ALERT_2_A Check Calcd Resid. Dens. 0.91A From Tl1 -3.85 eA-3
PLAT972_ALERT_2_A Check Calcd Resid. Dens. 1.42A From O4 -3.70 eA-3
PLAT972_ALERT_2_A Check Calcd Resid. Dens. 1.53A From O3 -3.59 eA-3
PLAT973_ALERT_2_A Check Calcd Positive Resid. Density on Tl2 4.34 eA-3
PLAT973_ALERT_2_A Check Calcd Positive Resid. Density on Tl1 3.97 eA-3
PLAT975_ALERT_2_A Check Calcd Resid. Dens. 1.06A From O6 2.68 eA-3

Alert level B

PLAT911_ALERT_3_B Missing FCF Refl Between Thmin & STh/L= 0.600 245 Report
PLAT971_ALERT_2_B Check Calcd Resid. Dens. 1.14A From Tl2 3.32 eA-3
PLAT971_ALERT_2_B Check Calcd Resid. Dens. 1.12A From Tl1 3.15 eA-3
PLAT971_ALERT_2_B Check Calcd Resid. Dens. 1.84A From Tl1 3.15 eA-3
PLAT971_ALERT_2_B Check Calcd Resid. Dens. 1.21A From Tl1 3.06 eA-3
PLAT971_ALERT_2_B Check Calcd Resid. Dens. 1.44A From Tl2 3.02 eA-3
PLAT971_ALERT_2_B Check Calcd Resid. Dens. 1.10A From Tl2 2.95 eA-3
PLAT971_ALERT_2_B Check Calcd Resid. Dens. 1.48A From O4 2.77 eA-3
PLAT971_ALERT_2_B Check Calcd Resid. Dens. 1.31A From O3 2.73 eA-3
PLAT971_ALERT_2_B Check Calcd Resid. Dens. 2.08A From F4 2.72 eA-3
PLAT971_ALERT_2_B Check Calcd Resid. Dens. 1.49A From O6 2.71 eA-3
PLAT971_ALERT_2_B Check Calcd Resid. Dens. 1.06A From O6 2.68 eA-3
PLAT971_ALERT_2_B Check Calcd Resid. Dens. 1.82A From F4 2.59 eA-3
PLAT972_ALERT_2_B Check Calcd Resid. Dens. 0.84A From Tl2 -3.41 eA-3
PLAT972_ALERT_2_B Check Calcd Resid. Dens. 1.41A From O6 -3.29 eA-3
PLAT972_ALERT_2_B Check Calcd Resid. Dens. 1.79A From Tl2 -3.17 eA-3
PLAT972_ALERT_2_B Check Calcd Resid. Dens. 1.66A From Tl1 -2.91 eA-3
PLAT972_ALERT_2_B Check Calcd Resid. Dens. 1.37A From Tl2 -2.88 eA-3
PLAT972_ALERT_2_B Check Calcd Resid. Dens. 1.32A From Tl2 -2.77 eA-3
PLAT972_ALERT_2_B Check Calcd Resid. Dens. 1.57A From O5 -2.67 eA-3
PLAT972_ALERT_2_B Check Calcd Resid. Dens. 1.36A From Tl2 -2.60 eA-3
PLAT972_ALERT_2_B Check Calcd Resid. Dens. 1.94A From F6 -2.56 eA-3
PLAT972_ALERT_2_B Check Calcd Resid. Dens. 1.72A From O3 -2.56 eA-3
PLAT972_ALERT_2_B Check Calcd Resid. Dens. 1.27A From Tl1 -2.54 eA-3

Alert level C

```
PLAT906_ALERT_3_C Large K Value in the Analysis of Variance ......      4.692 Check
PLAT971_ALERT_2_C Check Calcd Resid. Dens.  1.68A   From Tl2             2.50 eA-3
PLAT972_ALERT_2_C Check Calcd Resid. Dens.  1.26A   From O1             -2.49 eA-3
PLAT972_ALERT_2_C Check Calcd Resid. Dens.  1.55A   From O3             -2.47 eA-3
PLAT972_ALERT_2_C Check Calcd Resid. Dens.  1.31A   From O2             -2.46 eA-3
PLAT972_ALERT_2_C Check Calcd Resid. Dens.  1.26A   From Tl1            -2.41 eA-3
PLAT972_ALERT_2_C Check Calcd Resid. Dens.  1.51A   From O3             -2.41 eA-3
```

Alert level G

```
PLAT004_ALERT_5_G Polymeric Structure Found with Maximum Dimension          2 Info
PLAT040_ALERT_1_G No H-atoms in this Carbon Containing Compound ..     Please Check
PLAT072_ALERT_2_G SHELXL First  Parameter in WGHT  Unusually Large       0.13 Report
PLAT794_ALERT_5_G Tentative Bond Valency for Tl1       (I)       .       0.88 Info
PLAT794_ALERT_5_G Tentative Bond Valency for Tl2       (I)       .       0.92 Info
PLAT912_ALERT_4_G Missing # of FCF Reflections Above STh/L=  0.600        733 Note
PLAT941_ALERT_3_G Average HKL Measurement Multiplicity ...........        1.3 Low
```

```
  23 ALERT level A = Most likely a serious problem - resolve or explain
  24 ALERT level B = A potentially serious problem, consider carefully
   7 ALERT level C = Check. Ensure it is not caused by an omission or oversight
   7 ALERT level G = General information/check it is not something unexpected

   1 ALERT type 1 CIF construction/syntax error, inconsistent or missing data
  52 ALERT type 2 Indicator that the structure model may be wrong or deficient
   4 ALERT type 3 Indicator that the structure quality may be low
   1 ALERT type 4 Improvement, methodology, query or suggestion
   3 ALERT type 5 Informative message, check
```

Datablock: tlotf-htrop

```
Bond precision:   C-C = 0.0051 A           Wavelength=0.71073

Cell:            a=6.39693(11)     b=24.2622(4)        c=7.73214(15)
                 alpha=90          beta=108.917(2)     gamma=90
Temperature:     100 K
```

	Calculated	Reported
Volume	1135.24(4)	1135.24(4)
Space group	P 21/c	P 1 21/c 1
Hall group	-P 2ybc	-P 2ybc
Moiety formula	C8 H6 F3 O5 S Tl	C7 H6 O2 Tl, C F3 O3 S
Sum formula	C8 H6 F3 O5 S Tl	C8 H6 F3 O5 S Tl
Mr	475.57	475.56
Dx,g cm-3	2.783	2.782
Z	4	4
Mu (mm-1)	14.462	14.462
F000	872.0	872.0
F000'	862.30	
h,k,lmax	7,28,9	7,28,9
Nref	2001	1999
Tmin,Tmax	0.192,0.561	0.620,1.000
Tmin'	0.162	

Correction method= # Reported T Limits: Tmin=0.620 Tmax=1.000
AbsCorr = MULTI-SCAN

Data completeness= 0.999 Theta(max)= 24.991

R(reflections)= 0.0149(1884) wR2(reflections)= 0.0311(1999)

S = 1.092 Npar= 166

The following ALERTS were generated. Each ALERT has the format
test-name_ALERT_alert-type_alert-level.
Click on the hyperlinks for more details of the test.

Alert level G

PLAT004_ALERT_5_G	Polymeric Structure Found with Maximum Dimension	2 Info
PLAT042_ALERT_1_G	Calc. and Reported Moiety Formula Strings Differ	Please Check
PLAT794_ALERT_5_G	Tentative Bond Valency for Tl1 (I) .	0.82 Info
PLAT909_ALERT_3_G	Percentage of I>2sig(I) Data at Theta(Max) Still	86% Note
PLAT910_ALERT_3_G	Missing # of FCF Reflection(s) Below Theta(Min).	1 Note
PLAT941_ALERT_3_G	Average HKL Measurement Multiplicity	3.8 Low
PLAT978_ALERT_2_G	Number C-C Bonds with Positive Residual Density.	0 Info

---

0 **ALERT level A** = Most likely a serious problem - resolve or explain
0 **ALERT level B** = A potentially serious problem, consider carefully
0 **ALERT level C** = Check. Ensure it is not caused by an omission or oversight
7 **ALERT level G** = General information/check it is not something unexpected

1 ALERT type 1 CIF construction/syntax error, inconsistent or missing data

1 ALERT type 2 Indicator that the structure model may be wrong or deficient
3 ALERT type 3 Indicator that the structure quality may be low
0 ALERT type 4 Improvement, methodology, query or suggestion
2 ALERT type 5 Informative message, check

Datablock: tltrop

Bond precision: C-C = 0.0057 A Wavelength=0.71073

Cell:	a=7.39897(7)	b=13.10080(15)	c=7.37557(5)
	alpha=90	beta=90	gamma=90
Temperature:	100 K		

	Calculated	Reported
Volume	714.932(12)	714.932(12)
Space group	P c c a	P c c a
Hall group	-P 2a 2ac	-P 2a 2ac
Moiety formula	C7 H5 O2 Tl	C7 H5 O2 Tl
Sum formula	C7 H5 O2 Tl	C7 H5 O2 Tl
Mr	325.49	325.48
Dx,g cm-3	3.024	3.024
Z	4	4
Mu (mm-1)	22.519	22.519
F000	576.0	576.0
F000'	565.90	
h,k,lmax	10,18,10	10,18,10
Nref	1000	999
Tmin,Tmax	0.131,0.509	0.300,1.000
Tmin'	0.084	

Correction method= # Reported T Limits: Tmin=0.300 Tmax=1.000
AbsCorr = MULTI-SCAN

Data completeness= 0.999 Theta(max)= 29.498

R(reflections)= 0.0191(813) wR2(reflections)= 0.0578(999)

S = 1.070 Npar= 47

The following ALERTS were generated. Each ALERT has the format
test-name_ALERT_alert-type_alert-level.
Click on the hyperlinks for more details of the test.

Alert level C

```
PLAT906_ALERT_3_C Large K Value in the Analysis of Variance ......      5.403 Check
PLAT975_ALERT_2_C Check Calcd Resid. Dens.  1.04A   From O1              0.49 eA-3
PLAT975_ALERT_2_C Check Calcd Resid. Dens.  1.10A   From O1              0.42 eA-3
```

Alert level G

```
PLAT004_ALERT_5_G Polymeric Structure Found with Maximum Dimension          2 Info
PLAT143_ALERT_4_G s.u. on c - Axis Small or Missing .............     0.00005 Ang.
PLAT794_ALERT_5_G Tentative Bond Valency for Tl1       (I)       .       1.03 Info
PLAT910_ALERT_3_G Missing # of FCF Reflection(s) Below Theta(Min).          1 Note
PLAT978_ALERT_2_G Number C-C Bonds with Positive Residual Density.          2 Info
```

---

0 **ALERT level A** = Most likely a serious problem - resolve or explain
0 **ALERT level B** = A potentially serious problem, consider carefully
3 **ALERT level C** = Check. Ensure it is not caused by an omission or oversight
5 **ALERT level G** = General information/check it is not something unexpected

0 ALERT type 1 CIF construction/syntax error, inconsistent or missing data
3 ALERT type 2 Indicator that the structure model may be wrong or deficient
2 ALERT type 3 Indicator that the structure quality may be low
1 ALERT type 4 Improvement, methodology, query or suggestion
2 ALERT type 5 Informative message, check

Datablock: tlmetrop

Bond precision: C-C = 0.0186 A Wavelength=0.71073

Cell:	a=14.7373(8)	b=3.84328(15)	c=13.9390(6)
	alpha=90	beta=107.022(5)	gamma=90
Temperature:	100 K		

	Calculated	Reported
Volume	754.91(6)	754.91(6)
Space group	C 2	C 1 2 1
Hall group	C 2y	C 2y
Moiety formula	C8 H7 O2 Tl	C8 H7 O2 Tl
Sum formula	C8 H7 O2 Tl	C8 H7 O2 Tl
Mr	339.52	339.51
Dx,g cm-3	2.987	2.987
Z	4	4
Mu (mm-1)	21.333	21.333
F000	608.0	608.0
F000'	597.84	
h,k,lmax	18,4,17	18,4,17
Nref	1566[905]	1567
Tmin,Tmax	0.374,0.527	0.254,1.000
Tmin'	0.039	

Correction method= # Reported T Limits: Tmin=0.254 Tmax=1.000
AbsCorr = MULTI-SCAN

Data completeness= 1.73/1.00 Theta(max)= 26.491

R(reflections)= 0.0299(1485) wR2(reflections)= 0.0711(1567)

S = 1.064 Npar= 95

The following ALERTS were generated. Each ALERT has the format
test-name_ALERT_alert-type_alert-level.
Click on the hyperlinks for more details of the test.

Alert level B

```
PLAT972_ALERT_2_B Check Calcd Resid. Dens.  0.96A   From Tl1           -3.45 eA-3
PLAT972_ALERT_2_B Check Calcd Resid. Dens.  0.88A   From Tl1           -3.20 eA-3
```

Alert level C

```
PLAT213_ALERT_2_C Atom C6              has ADP max/min Ratio .....         4.0 oblate
PLAT250_ALERT_2_C Large U3/U1 Ratio for Average U(i,j) Tensor ....         2.3 Note
PLAT342_ALERT_3_C Low Bond Precision on  C-C Bonds ..............     0.01863 Ang.
PLAT975_ALERT_2_C Check Calcd Resid. Dens.  0.88A   From O2             0.53 eA-3
PLAT975_ALERT_2_C Check Calcd Resid. Dens.  0.88A   From O2             0.53 eA-3
PLAT977_ALERT_2_C Check Negative Difference Density on H8B            -0.34 eA-3
```

Alert level G

```
PLAT004_ALERT_5_G Polymeric Structure Found with Maximum Dimension           1 Info
PLAT171_ALERT_4_G The CIF-Embedded .res File Contains EADP Records           1 Report
```

```
PLAT794_ALERT_5_G Tentative Bond Valency for Tl1       (I)       .       1.10 Info
PLAT910_ALERT_3_G Missing # of FCF Reflection(s) Below Theta(Min).          1 Note
PLAT941_ALERT_3_G Average HKL Measurement Multiplicity ..........         3.5 Low
PLAT978_ALERT_2_G Number C-C Bonds with Positive Residual Density.          0 Info
```

---

0 **ALERT level A** = Most likely a serious problem - resolve or explain
2 **ALERT level B** = A potentially serious problem, consider carefully
6 **ALERT level C** = Check. Ensure it is not caused by an omission or oversight
6 **ALERT level G** = General information/check it is not something unexpected

0 ALERT type 1 CIF construction/syntax error, inconsistent or missing data
8 ALERT type 2 Indicator that the structure model may be wrong or deficient
3 ALERT type 3 Indicator that the structure quality may be low
1 ALERT type 4 Improvement, methodology, query or suggestion
2 ALERT type 5 Informative message, check

Datablock: tlhino

Bond precision: C-C = 0.0067 A Wavelength=1.54184

Cell:	a=30.3085(3)	b=7.6331(1)	c=35.5281(4)
	alpha=90	beta=101.171(1)	gamma=90
Temperature:	100 K		

	Calculated	Reported
Volume	8063.62(16)	8063.62(16)
Space group	C 2/c	C 1 2/c 1
Hall group	-C 2yc	-C 2yc
Moiety formula	C40 H44 O8 Tl4	C40 H44 O8 Tl4
Sum formula	C40 H44 O8 Tl4	C40 H44 O8 Tl4
Mr	1470.27	1470.23
Dx,g cm-3	2.422	2.422
Z	8	8
Mu (mm-1)	30.523	30.523
F000	5376.0	5376.0
F000’	5256.18	
h,k,lmax	36,9,42	36,9,42
Nref	7447	7413
Tmin,Tmax	0.167,0.400	0.057,1.000
Tmin’	0.003	

Correction method= # Reported T Limits: Tmin=0.057 Tmax=1.000
AbsCorr = MULTI-SCAN

Data completeness= 0.995 Theta(max)= 68.709

R(reflections)= 0.0237(7243) wR2(reflections)= 0.0589(7413)

S = 1.220 Npar= 477

The following ALERTS were generated. Each ALERT has the format
test-name_ALERT_alert-type_alert-level.
Click on the hyperlinks for more details of the test.

Alert level C

```
PLAT906_ALERT_3_C Large K Value in the Analysis of Variance ......      2.139 Check
PLAT911_ALERT_3_C Missing FCF Refl Between Thmin & STh/L=    0.600          3 Report
PLAT975_ALERT_2_C Check Calcd Resid. Dens.  0.81A   From O11             0.62 eA-3
```

Alert level G

```
PLAT004_ALERT_5_G Polymeric Structure Found with Maximum Dimension          1 Info
PLAT083_ALERT_2_G SHELXL Second Parameter in WGHT  Unusually Large      34.90 Why ?
PLAT794_ALERT_5_G Tentative Bond Valency for Tl1       (I)       .       0.99 Info
PLAT794_ALERT_5_G Tentative Bond Valency for Tl2       (I)       .       1.06 Info
PLAT794_ALERT_5_G Tentative Bond Valency for Tl3       (I)       .       1.05 Info
PLAT794_ALERT_5_G Tentative Bond Valency for Tl4       (I)       .       1.03 Info
PLAT912_ALERT_4_G Missing # of FCF Reflections Above STh/L=  0.600         32 Note
PLAT941_ALERT_3_G Average HKL Measurement Multiplicity ..........        4.6 Low
PLAT978_ALERT_2_G Number C-C Bonds with Positive Residual Density.          0 Info
```

---

0 **ALERT level A** = Most likely a serious problem - resolve or explain
0 **ALERT level B** = A potentially serious problem, consider carefully
3 **ALERT level C** = Check. Ensure it is not caused by an omission or oversight
9 **ALERT level G** = General information/check it is not something unexpected

0 ALERT type 1 CIF construction/syntax error, inconsistent or missing data
3 ALERT type 2 Indicator that the structure model may be wrong or deficient
3 ALERT type 3 Indicator that the structure quality may be low
1 ALERT type 4 Improvement, methodology, query or suggestion
5 ALERT type 5 Informative message, check

Datablock: tl7hino6otf

Bond precision: C-C = 0.0118 A Wavelength=0.71073

Cell:	a=19.4523(3)	b=18.26435(15)	c=20.5764(3)
	alpha=90	beta=116.2430(17)	gamma=90
Temperature:	100 K		

	Calculated	Reported
Volume	6556.95(18)	6556.95(16)
Space group	P 21/c	P 1 21/c 1
Hall group	-P 2ybc	-P 2ybc
Moiety formula	C60 H66 O12 Tl7, C F3 O3 S	3(C20 H22 O4 Tl2), Tl, C F3 O3 S
Sum formula	C61 H66 F3 O15 S Tl7	C61 H66 F3 O15 S Tl7
Mr	2558.86	2558.78
Dx,g cm-3	2.592	2.592
Z	4	4
Mu (mm-1)	17.241	17.241
F000	4648.0	4648.0
F000'	4576.95	
h,k,lmax	24,23,26	24,23,26
Nref	14320	14315
Tmin,Tmax	0.042,0.596	0.197,1.000
Tmin'	0.011	

Correction method= # Reported T Limits: Tmin=0.197 Tmax=1.000
AbsCorr = MULTI-SCAN

Data completeness= 1.000 Theta(max)= 27.000

R(reflections)= 0.0346(11747) wR2(reflections)= 0.0754(14315)

S = 1.078 Npar= 843

The following ALERTS were generated. Each ALERT has the format
test-name_ALERT_alert-type_alert-level.
Click on the hyperlinks for more details of the test.

Alert level C

```
PLAT213_ALERT_2_C Atom C7              has ADP max/min Ratio .....        3.1 oblate
PLAT213_ALERT_2_C Atom C13             has ADP max/min Ratio .....        3.5 oblate
PLAT213_ALERT_2_C Atom C51             has ADP max/min Ratio .....        3.8 prolat
PLAT214_ALERT_2_C Atom F3A       (Anion/Solvent) ADP max/min Ratio        4.4 prolat
PLAT214_ALERT_2_C Atom O3A       (Anion/Solvent) ADP max/min Ratio        4.9 prolat
PLAT214_ALERT_2_C Atom O4A       (Anion/Solvent) ADP max/min Ratio        4.1 prolat
PLAT214_ALERT_2_C Atom F2B       (Anion/Solvent) ADP max/min Ratio        4.5 prolat
PLAT220_ALERT_2_C NonSolvent   Resd 1  C   Ueq(max)/Ueq(min) Range        3.5 Ratio
PLAT222_ALERT_3_C NonSolvent Resd 1  H   Uiso(max)/Uiso(min) Range        4.1 Ratio
PLAT244_ALERT_4_C Low    'Solvent' Ueq as Compared to Neighbors of        S1 Check
PLAT342_ALERT_3_C Low Bond Precision on  C-C Bonds ..............     0.01182 Ang.
PLAT910_ALERT_3_C Missing # of FCF Reflection(s) Below Theta(Min).          5 Note
PLAT971_ALERT_2_C Check Calcd Resid. Dens.  1.48A   From Tl6           1.56 eA-3
PLAT971_ALERT_2_C Check Calcd Resid. Dens.  1.33A   From Tl7           1.52 eA-3
PLAT971_ALERT_2_C Check Calcd Resid. Dens.  1.22A   From Tl3           1.51 eA-3
PLAT975_ALERT_2_C Check Calcd Resid. Dens.  0.76A   From O52           1.15 eA-3
```

```
PLAT977_ALERT_2_C Check Negative Difference Density on H16            -0.49 eA-3
PLAT977_ALERT_2_C Check Negative Difference Density on H19B           -0.43 eA-3
PLAT977_ALERT_2_C Check Negative Difference Density on H40B           -0.33 eA-3
PLAT977_ALERT_2_C Check Negative Difference Density on H57            -0.52 eA-3
```

Alert level G

```
PLAT004_ALERT_5_G Polymeric Structure Found with Maximum Dimension          2 Info
PLAT042_ALERT_1_G Calc. and Reported Moiety Formula Strings Differ     Please Check
PLAT083_ALERT_2_G SHELXL Second Parameter in WGHT  Unusually Large      24.95 Why ?
PLAT142_ALERT_4_G s.u. on b - Axis Small or Missing .............     0.00015 Ang.
PLAT152_ALERT_1_G The Supplied and Calc. Volume s.u. Differ by ...          2 Units
PLAT244_ALERT_4_G Low    'Solvent' Ueq as Compared to Neighbors of        C61 Check
PLAT302_ALERT_4_G Anion/Solvent/Minor-Residue Disorder (Resd  2  )        63% Note
PLAT794_ALERT_5_G Tentative Bond Valency for Tl1       (I)       .       1.03 Info
PLAT794_ALERT_5_G Tentative Bond Valency for Tl2       (I)       .       1.00 Info
PLAT794_ALERT_5_G Tentative Bond Valency for Tl3       (I)       .       0.99 Info
PLAT794_ALERT_5_G Tentative Bond Valency for Tl4       (I)       .       0.94 Info
PLAT794_ALERT_5_G Tentative Bond Valency for Tl5       (I)       .       0.99 Info
PLAT794_ALERT_5_G Tentative Bond Valency for Tl6       (I)       .       0.98 Info
PLAT794_ALERT_5_G Tentative Bond Valency for Tl7       (I)       .       0.73 Info
PLAT913_ALERT_3_G Missing # of Very Strong Reflections in FCF ....          1 Note
PLAT978_ALERT_2_G Number C-C Bonds with Positive Residual Density.          0 Info
```

---

 0 **ALERT level A** = Most likely a serious problem - resolve or explain
 0 **ALERT level B** = A potentially serious problem, consider carefully
20 **ALERT level C** = Check. Ensure it is not caused by an omission or oversight
16 **ALERT level G** = General information/check it is not something unexpected

```
 2 ALERT type 1 CIF construction/syntax error, inconsistent or missing data
18 ALERT type 2 Indicator that the structure model may be wrong or deficient
 4 ALERT type 3 Indicator that the structure quality may be low
 4 ALERT type 4 Improvement, methodology, query or suggestion
 8 ALERT type 5 Informative message, check
```

It is advisable to attempt to resolve as many as possible of the alerts in all categories. Often the minor alerts point to easily fixed oversights, errors and omissions in your CIF or refinement strategy, so attention to these fine details can be worthwhile. In order to resolve some of the more serious problems it may be necessary to carry out additional measurements or structure refinements. However, the purpose of your study may justify the reported deviations and the more serious of these should normally be commented upon in the discussion or experimental section of a paper or in the "special_details" fields of the CIF. checkCIF was carefully designed to identify outliers and unusual parameters, but every test has its limitations and alerts that are not important in a particular case may appear. Conversely, the absence of alerts does not guarantee there are no aspects of the results needing attention. It is up to the individual to critically assess their own results and, if necessary, seek expert advice.

Publication of your CIF in IUCr journals

A basic structural check has been run on your CIF. These basic checks will be run on all CIFs submitted for publication in IUCr journals (*Acta Crystallographica*, *Journal of Applied Crystallography*, *Journal of Synchrotron Radiation*); however, if you intend to submit to *Acta Crystallographica Section C* or *E* or *IUCrData*, you should make sure that full publication checks are run on the final version of your CIF prior to submission.

Publication of your CIF in other journals

Please refer to the *Notes for Authors* of the relevant journal for any special instructions relating to CIF submission.

PLATON version of 13/07/2021; check.def file version of 13/07/2021

Datablock tlotf - ellipsoid plot

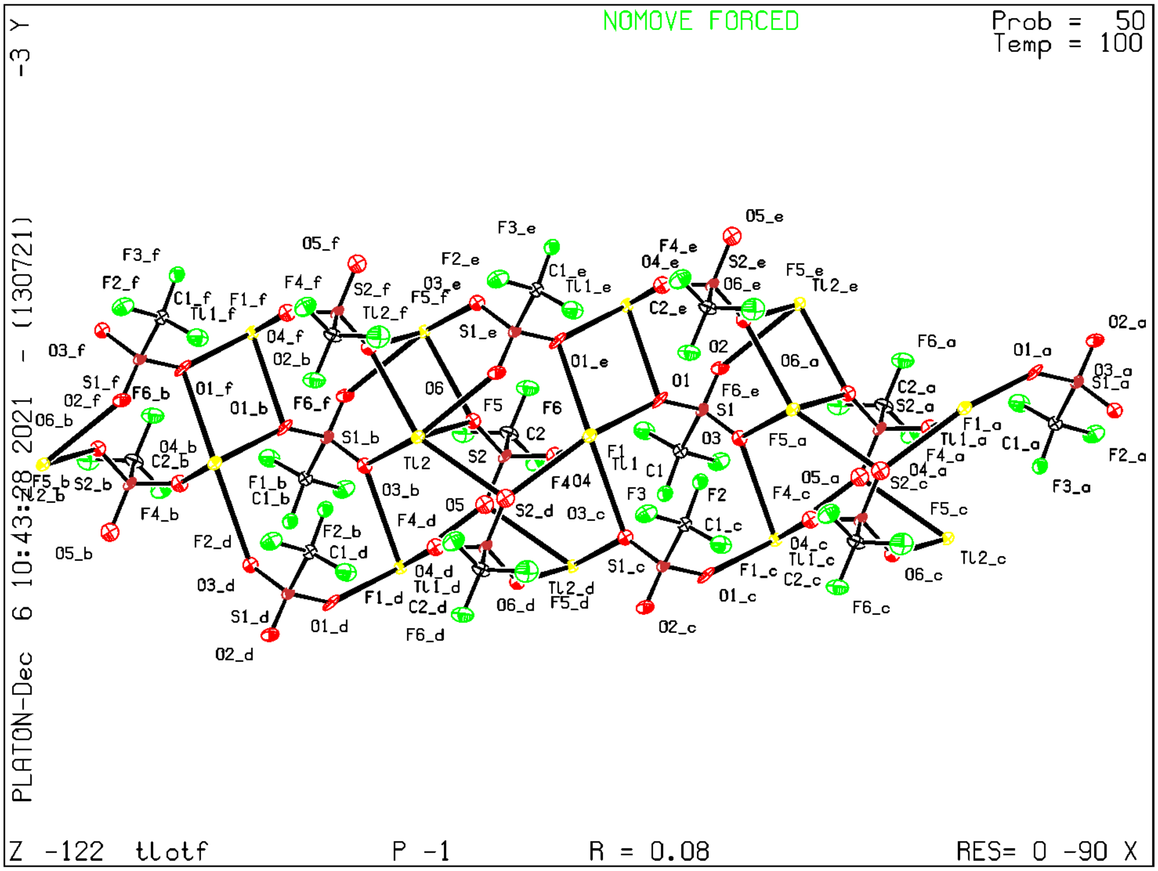

Datablock tlotf-htrop - ellipsoid plot

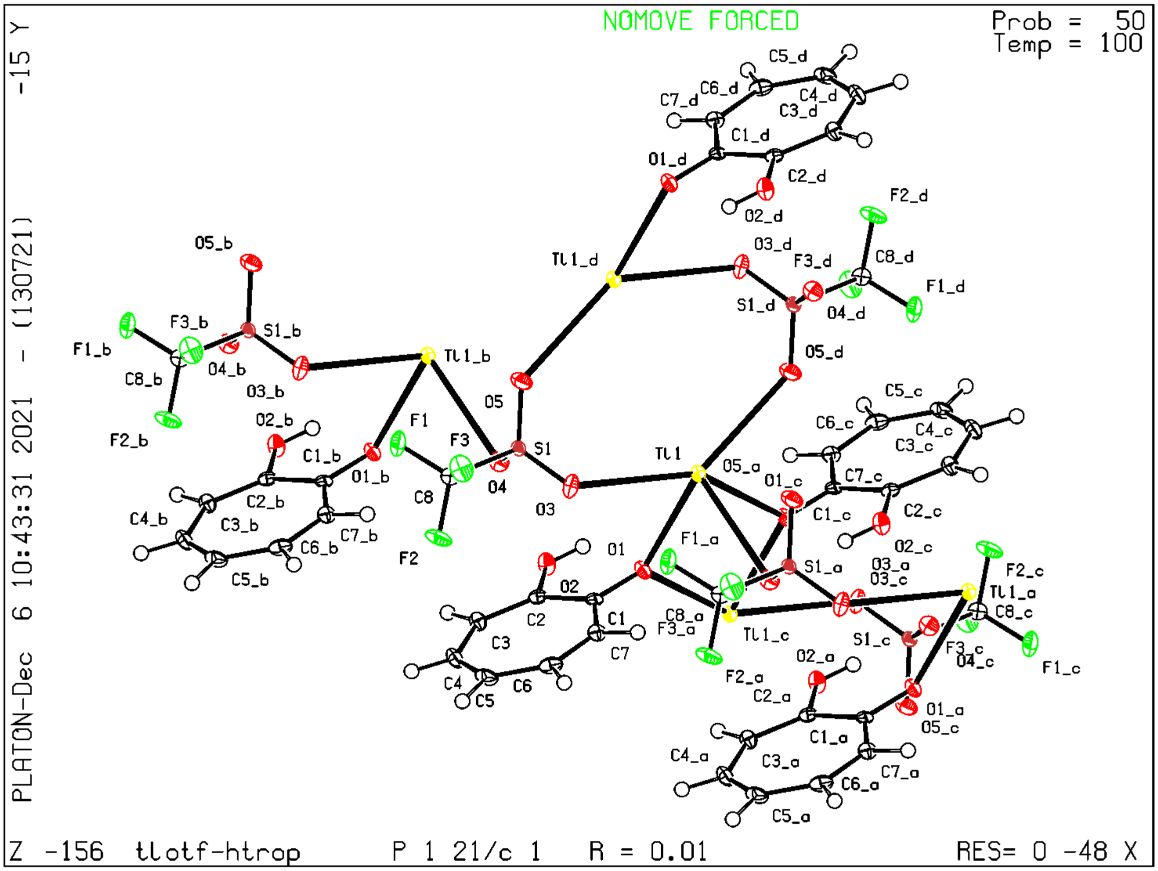

Datablock tltrop - ellipsoid plot

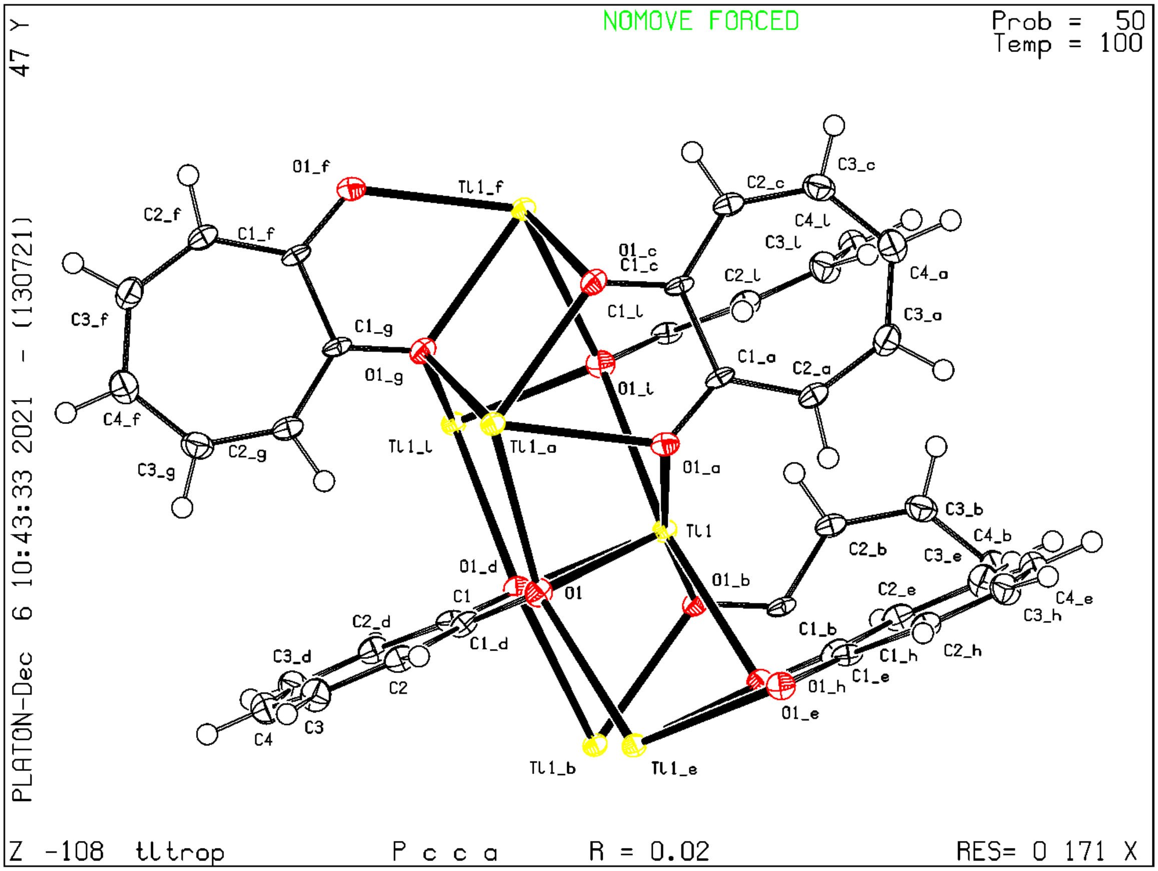

Datablock tlmetrop - ellipsoid plot

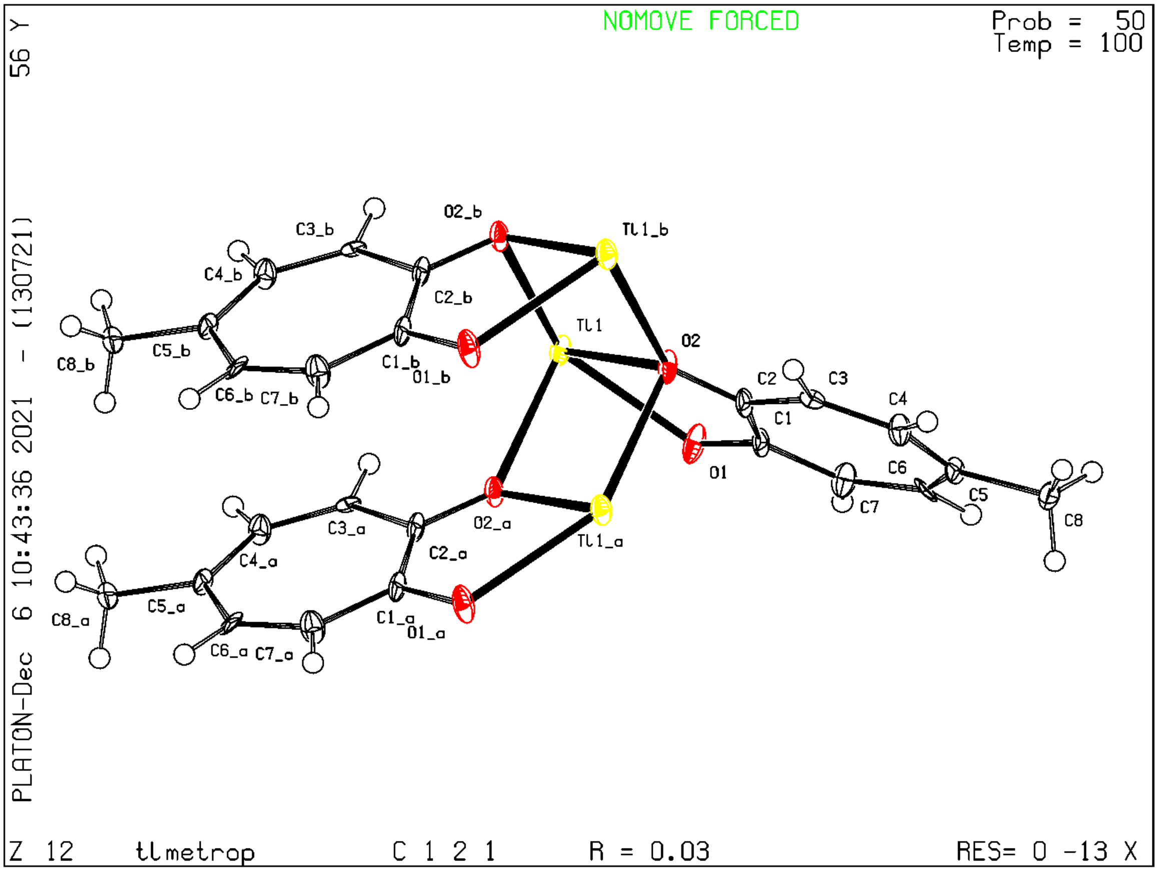

Datablock tlhino - ellipsoid plot

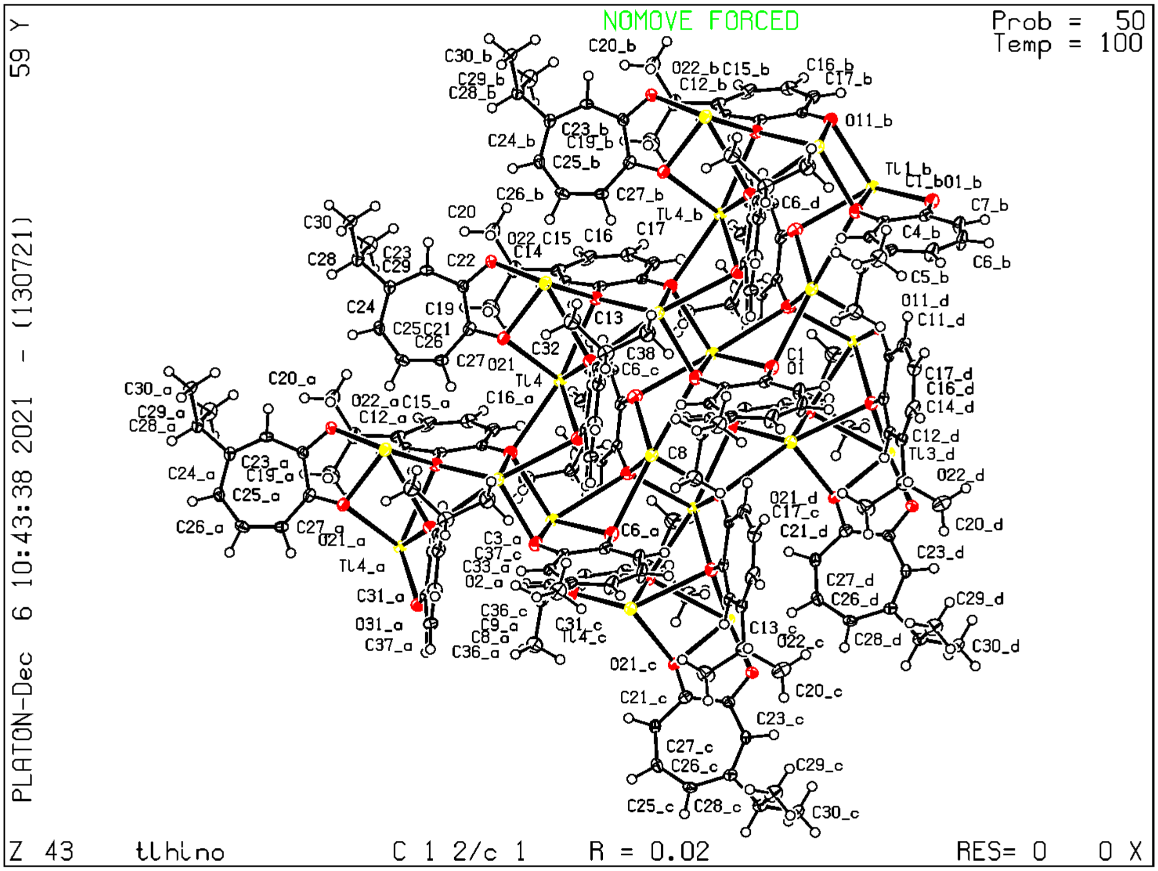

Datablock tl7hino6otf - ellipsoid plot

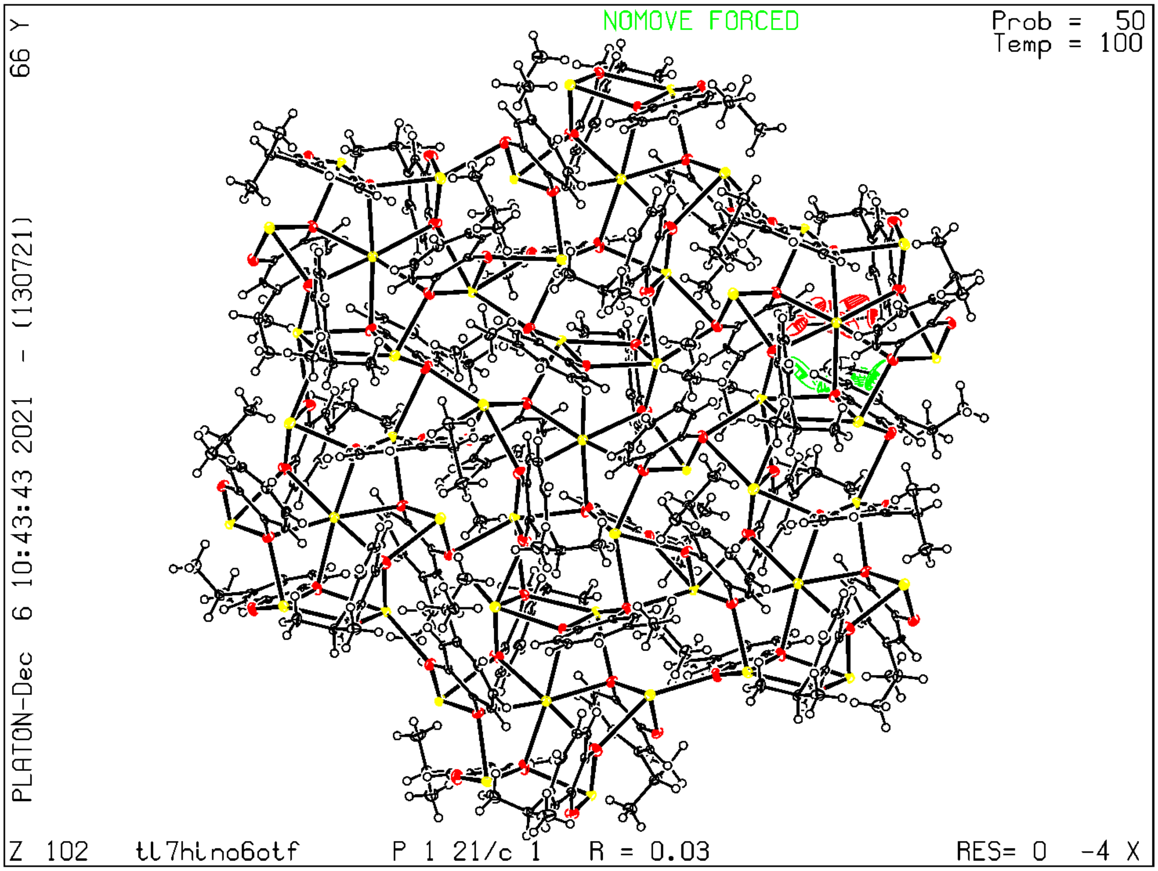

Supplement: Supplementary file 1 [file molecules-27-00183-s001.zip › CheckCif_KL.pdf]
