# Supplementary material for: Thallium(I) Tropolonates: Synthesis, Structure, Spectral Characteristics, and Antimicrobial Activity Compared to Lead(II) and Bismuth(III) Analogues
Source: Molecules. 2021 Dec 29;27(1):183. doi: 10.3390/molecules27010183 (PMC8746424; doi:10.3390/molecules27010183)
Supplement: Supplementary file 1 [file molecules-27-00183-s001.zip › Supplementary Material_KL_corrected.pdf]

Thallium(I) tropolonates: synthesis, structure, spectral characteristics, and antimicrobial activity compared to lead(II) and bismuth(III) analogues

Krzysztof Lyczko [1,], Monika Lyczko [1], Marzena Banasiewicz [2], Karolina Wegrzynska [3], Anna Ziółko [3], Anna Baraniak [3] and Jan Cz. Dobrowolski [1,3]*

[1] Institute of Nuclear Chemistry and Technology, Dorodna 16, 03-195 Warsaw, Poland; k.lyczko@ichtj.waw.pl (K.L.)

[2] Institute of Physics PAS, Al. Lotnikow 32/46, 02-668 Warsaw, Poland

[3] National Medicines Institute, Chełmska 30/34, 00-725 Warsaw, Poland

* Correspondence: k.lyczko@ichtj.waw.pl

Supplementary Materials

Table S1. Crystal data and structure refinement details for studied thallium(I) compounds.

Figure S1. ATR-FTIR of Tl(OTf).

Figure S2. ATR-FTIR of Tl(OTf)·Htrop adduct.

Figure S3. ATR-FTIR of Htrop.

Figure S4. ATR-FTIR of Tl(trop) (**1**).

Figure S5. ATR-FTIR of 5-meHtrop.

Figure S6. ATR-FTIR of Tl(5-metrop) (**2**).

Figure S7. ATR-FTIR of Hhino.

Figure S8. ATR-FTIR of Tl(hino) (**3**).

Figure S9. ATR-FTIR of {Tl@[Tl(hino)]$_6$}(OTf) (**4**).

Figure S10. Experimental UV-Vis absorption spectra of the studied thallium(I) complexes (red lines) in comparison with the respective ligand (black lines) measured in MeOH solutions.

Figure S11. Excitation and emission spectra for complexes **2**–**5** in methanol (left) and the solid state (right).

Figure S12. ^{13}C NMR spectrum of Tl(OTf).

Figure S13 a) ^{1}H NMR, **b)** ^{13}C NMR and **c)** ^{19}F NMR spectra of Tl(OTf)·Htrop adduct.

Figure S14 a) ^{1}H NMR and **b)** ^{13}C NMR spectra of Tl(trop) (**1**).

Figure S15 a) ^{1}H NMR and **b)** ^{13}C NMR spectra of Tl(5-metrop) (**2**).

Figure S16 a) ^{1}H NMR and **b)** ^{13}C NMR spectra of Tl(hino) (**3**).

Figure S17 a) ^{1}H NMR, **b)** ^{13}C NMR and **c)** ^{19}F NMR spectra of {Tl@[Tl(hino)]$_6$}(OTf) (**4**).

Figure S18. ^{205}Tl NMR spectra of studied thallium(I) compounds.

Table S1.

	Tl(OTf)	Tl(OTf)·Htrop	Tl(trop) (1)	Tl(5-metrop)	Tl(hino) (3)	{Tl[Tl(hino)]$_6$}(OTf) (4)
chemical formula	$C_2F_6O_6S_2Tl_2$	$C_8H_6F_3O_5STl$	$C_7H_5O_2Tl$	$C_8H_7O_2Tl$	$C_{40}H_{44}O_8Tl_4$	$C_{61}H_{66}F_3O_{15}STl_7$
formula weight	706.88	475.56	325.48	339.51	1470.23	2558.78
λ (Mo/Cu Kα) (Å)	0.71073	0.71073	0.71073	0.71073	1.54184	0.71073
crystal system	triclinic	monoclinic	orthorhombic	monoclinic	monoclinic	monoclinic
space group	$P\,\bar{1}$	$P\,2_1/c$	P c c a	C 2	C 2/c	$P\,2_1/c$
a (Å)	7.5368(15)	6.39693(11)	7.39897(7)	14.7373(8)	30.3085(3)	19.4523(3)
b (Å)	8.3722(18)	24.2622(4)	13.10080(15)	3.84328(15)	7.63310(10)	18.26435(15)
c (Å)	9.3994(19)	7.73214(15)	7.37557(5)	13.9390(6)	35.5281(4)	20.5764(3)
α (°)	88.170(17)	90.0	90.0	90.0	90.00	90.00
β (°)	73.168(18)	108.917(2)	90.0	107.022(5)	101.1710(10)	116.2430(17)
γ (°)	89.134(17)	90.0	90.0	90.0	90.00	90.00
V (Å^3)	567.4(2)	1135.24(3)	714.932(12)	754.91(6)	8063.62(16)	6556.95(16)
Z	2	4	4	4	8	4
$D_{calc.}$ (g·cm^{-3})	4.138	2.782	3.024	2.987	2.422	2.592
μ (mm^{-1})	28.830	14.462	22.519	21.333	30.523	17.241
F(000)	616	872	576	608	5376	4648
ϑ range (°)	2.3 – 31.2	2.9 – 25.0	3.1 – 29.5	2.8 – 26.5	2.5 – 68.7	2.2 – 27.0
crystal size (mm)	0.18 x 0.16 x	0.12 x 0.10 x	0.10 x 0.10 x	0.15 x 0.04 x	0.20 x 0.07 0.03	0.24 x 0.17 x 0.03
reflections collected	3389	7667	43165	3177	33889	84617
unique reflections	2684	1999	999	1567	7413	14315
reflections $I > 2\sigma(I)$	1953	1884	813	1485	7243	11747
R_{int}	0.0584	0.0218	0.0503	0.0384	0.0265	0.0703
restraints/parameters	0 / 138	0 / 166	0 / 47	1 / 95	0 / 477	0 / 843
goodness-of-fit	1.041	1.092	1.071	1.055	1.221	1.078
R_1, wR_2 ($I > 2\sigma(I)$)^a	0.0774, 0.1959	0.0149, 0.0307	0.0191, 0.0530	0.0299, 0.0699	0.0237, 0.0585	0.0346, 0.0689
R_1, wR_2 (all data)^a	0.1046, 0.2173	0.0169, 0.0312	0.0250, 0.0578	0.0314, 0.0712	0.0246, 0.0589	0.0484, 0.0754
largest diff. peak/hole (e·Å^{-3})	5.761 / –5.465	0.448 / –0.580	0.823 / –1.125	1.240 / –3.375	1.395 / –0.993	1.893 / –1.754

$^aR_1 = \Sigma|\,|F_o|-|F_c|\,|/\Sigma|F_o|$, $wR_2 = \{\Sigma[w(F_o^2 - F_c^2)^2]/\Sigma[w(F_o^2)^2]\}^{1/2}$

Figure S1.

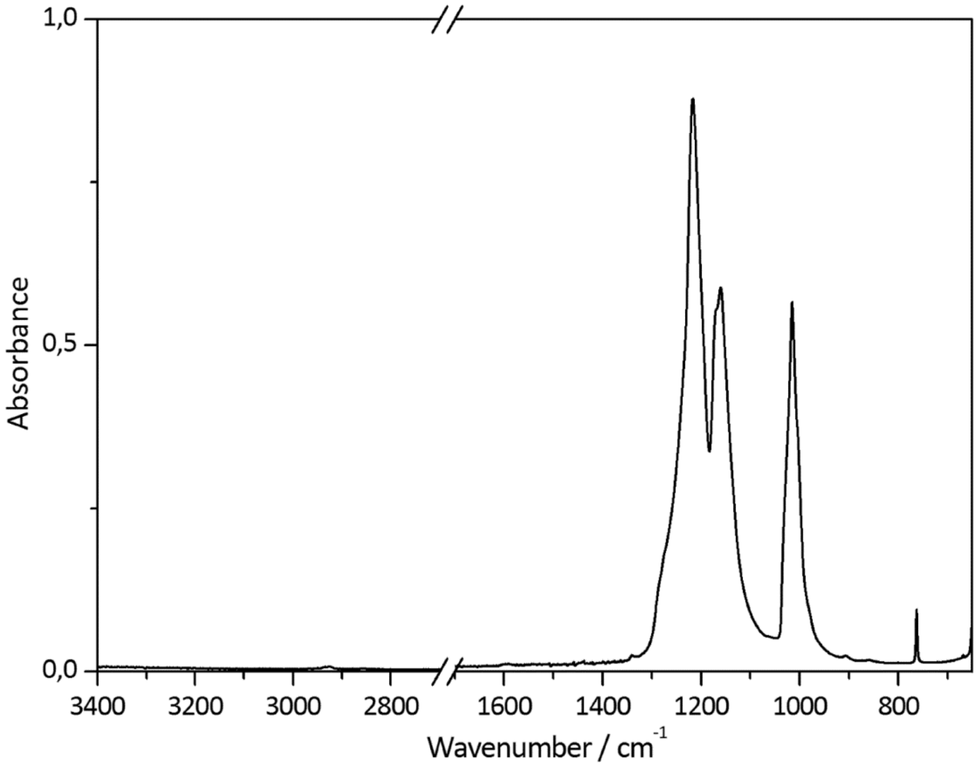

Figure S2.

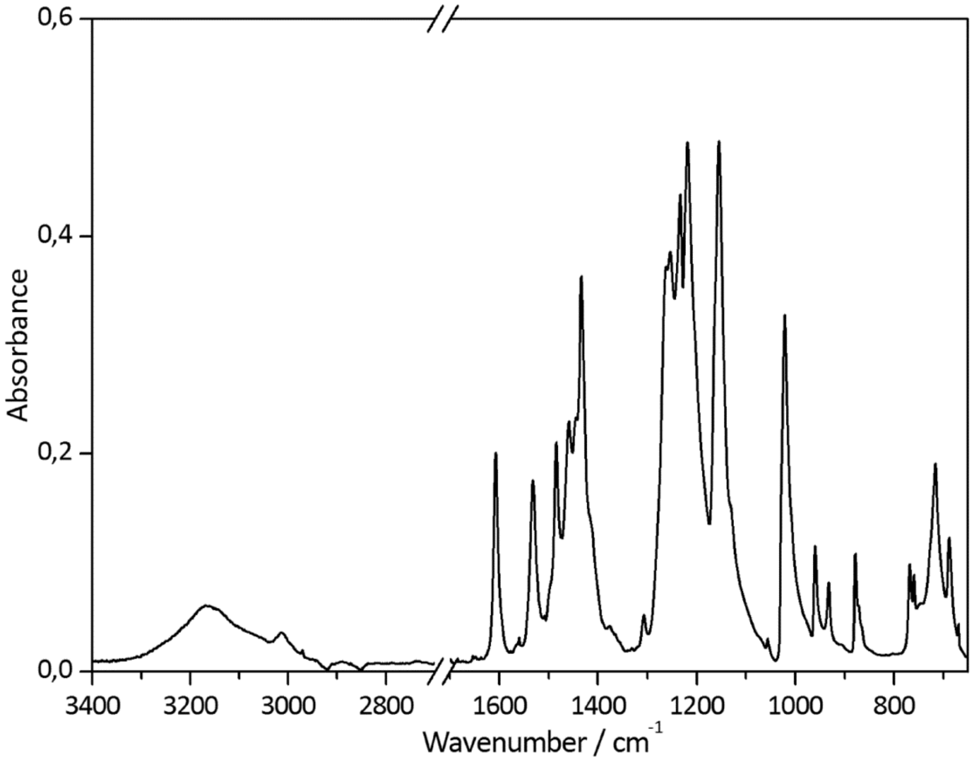

Figure S3.

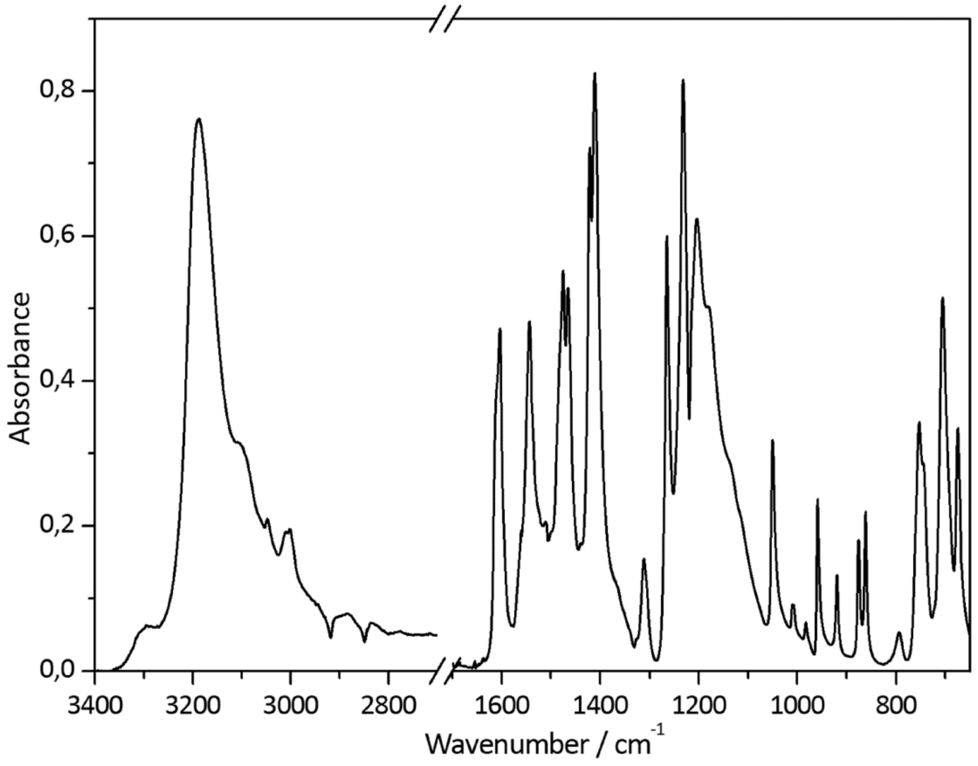

Figure S4.

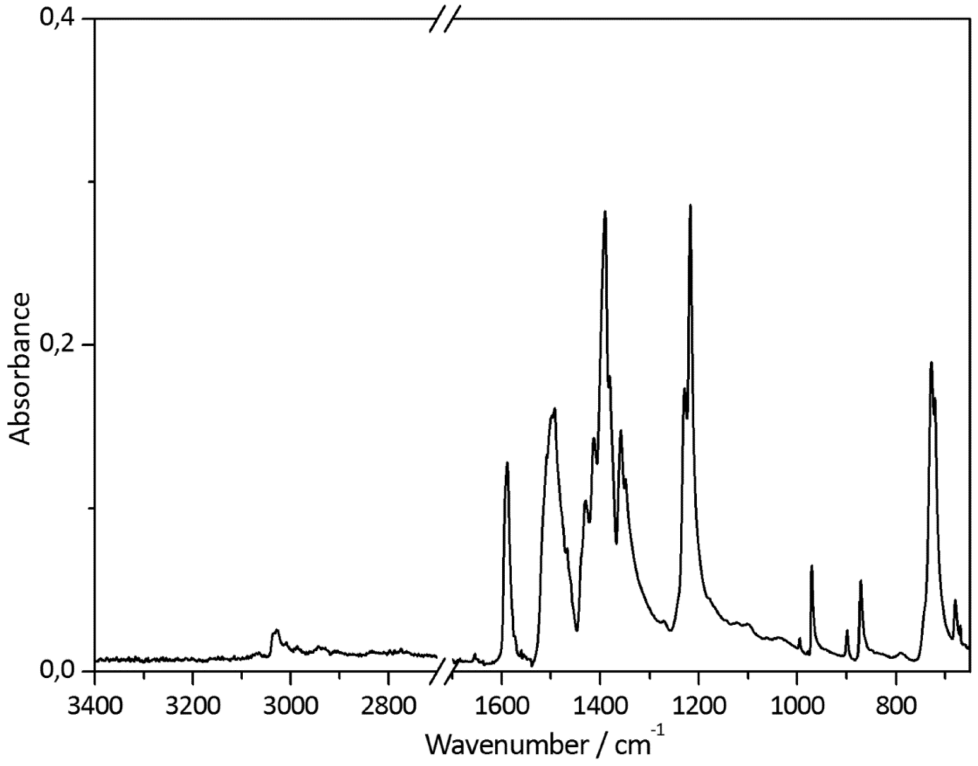

Figure S5.

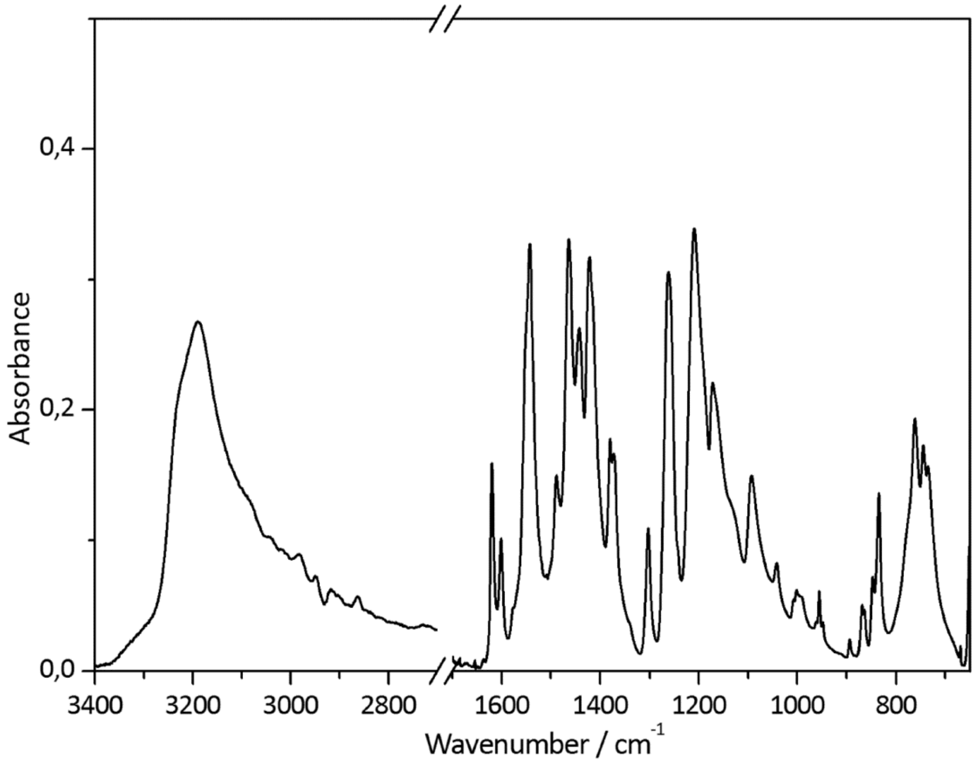

Figure S6.

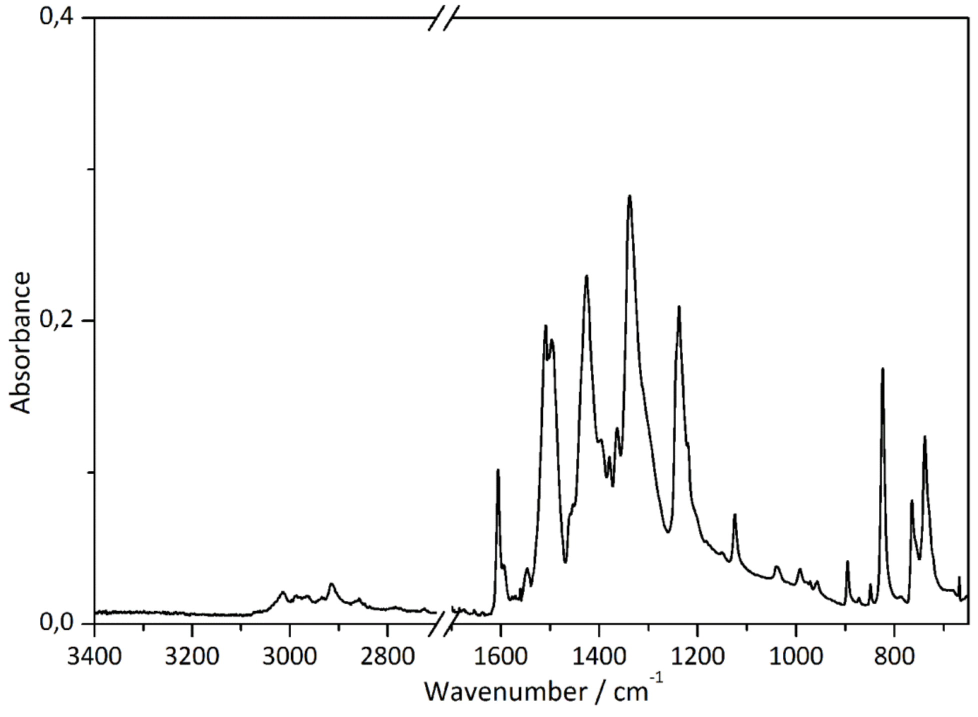

Figure S7.

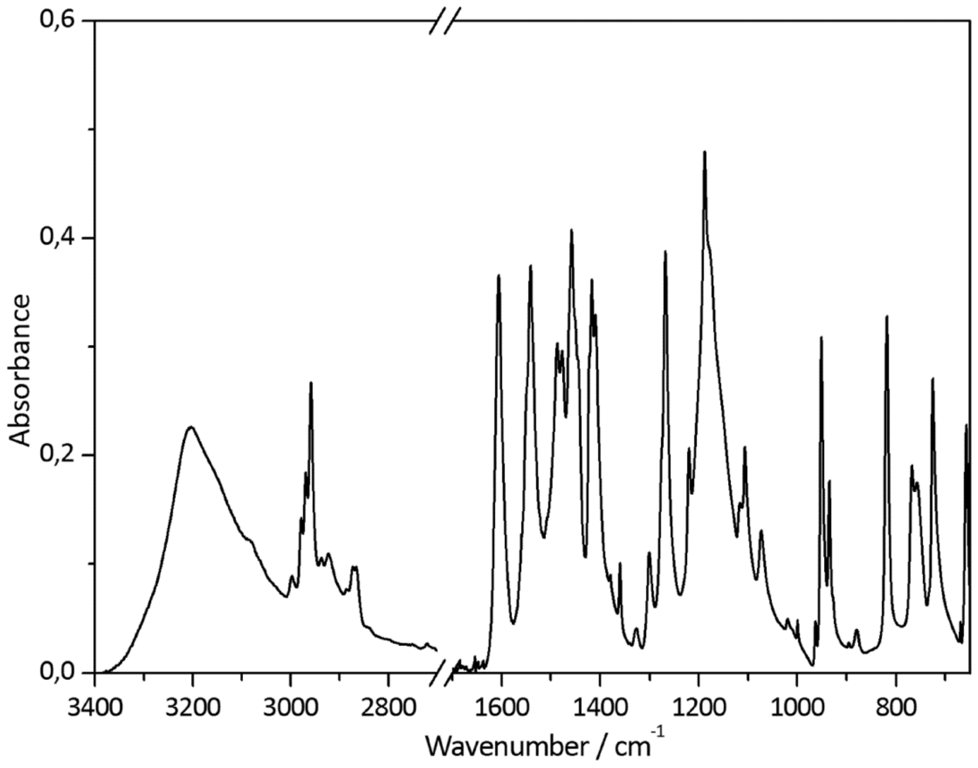

Figure S8.

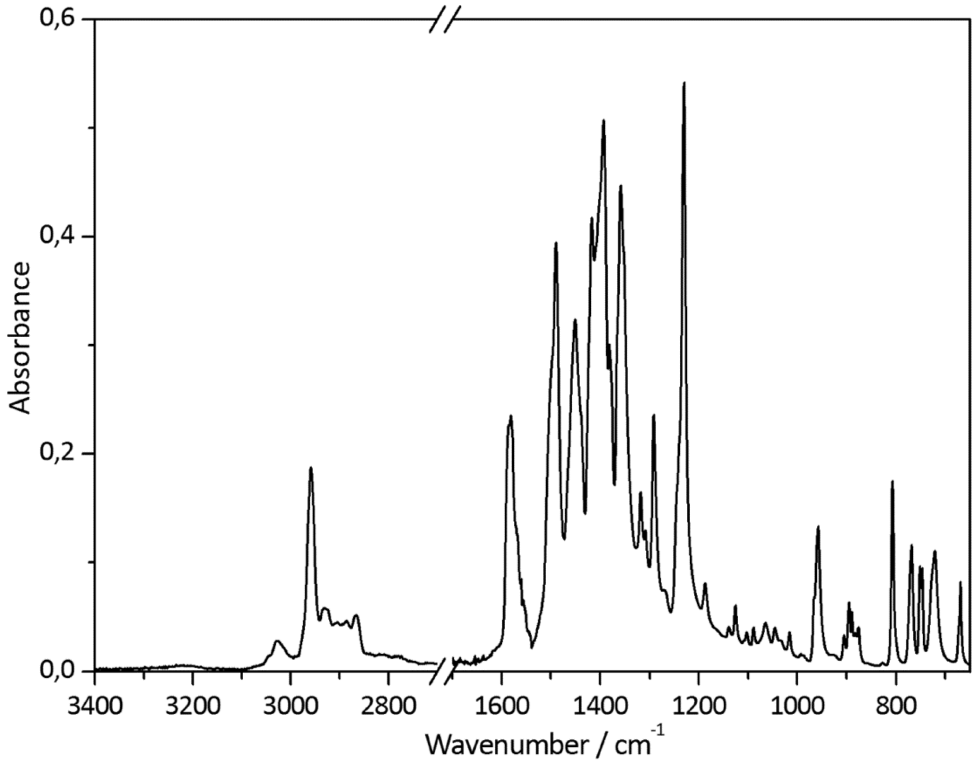

Figure S9.

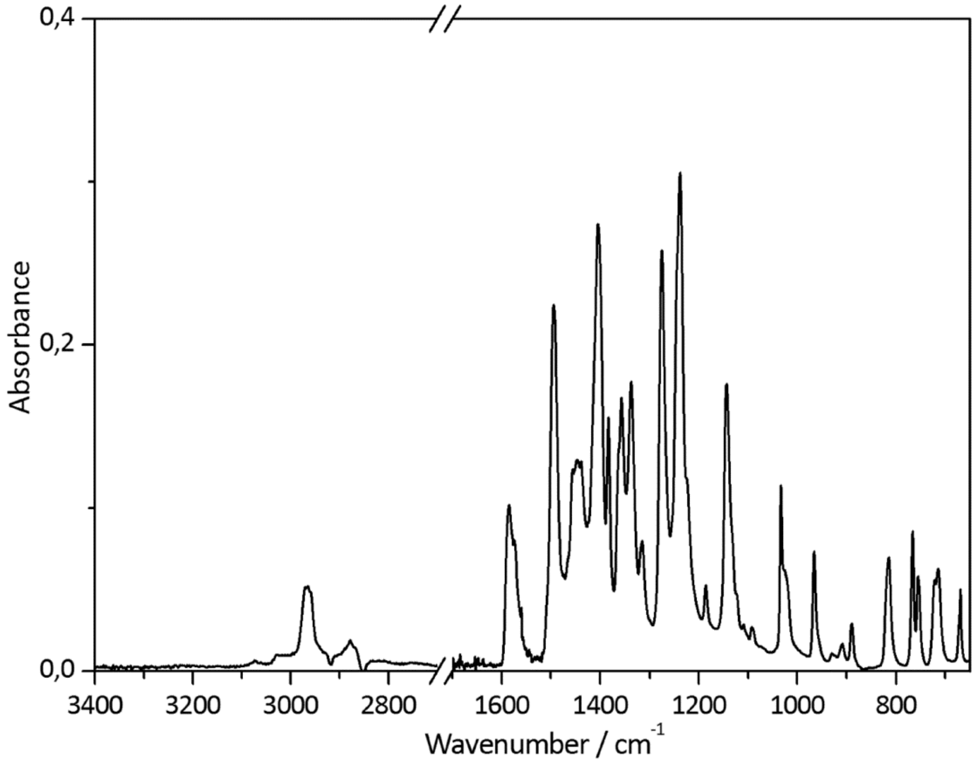
0,4
0,2
0,0
Absorbance
3400
3200
3000
2800
1600
1400
1200
1000
800
Wavenumber / cm-1

Figure S10.

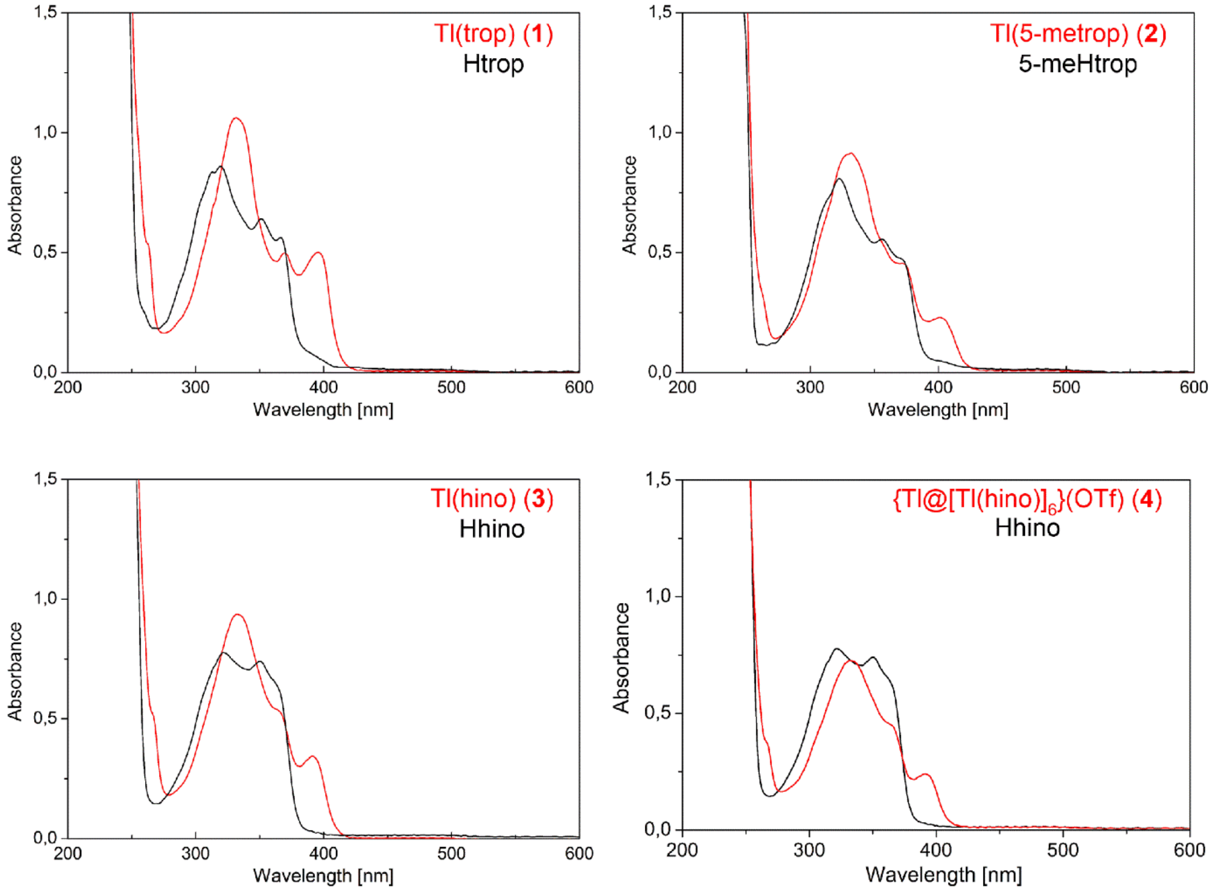
Tl(trop) (1)
Htrop
Tl(5-metrop) (2)
5-meHtrop
Tl(hino) (3)
Hhino
{Tl@[Tl(hino)]$_6$}(OTf) (4)
Hhino
Absorbance
Wavelength [nm]
0,0
0,5
1,0
1,5
200
300
400
500
600

Figure S11.

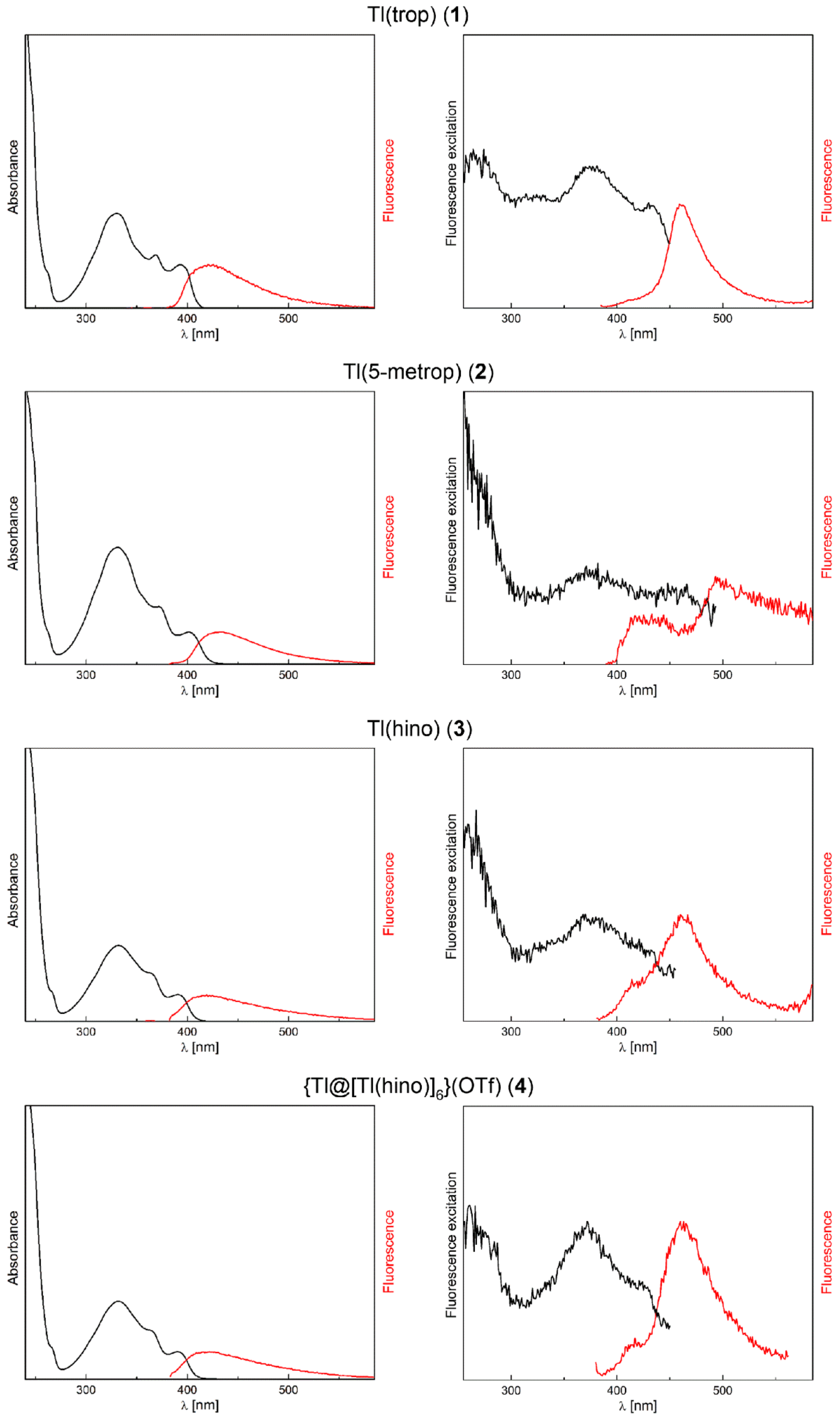
Tl(trop) (1)
Absorbance
Fluorescence
Fluorescence excitation
300
400
500
λ [nm]
Tl(5-metrop) (2)
Tl(hino) (3)
{Tl@[Tl(hino)]$_6$}(OTf) (4)

Figure S12.

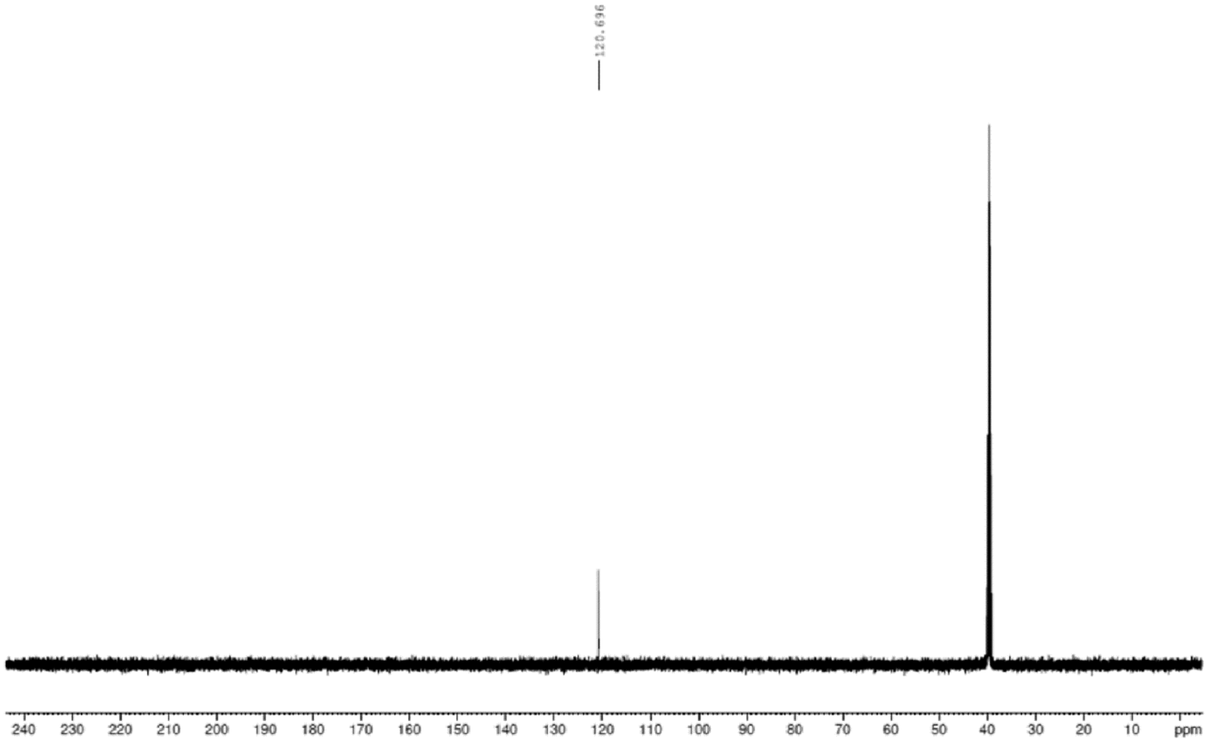

120.696
240 230 220 210 200 190 180 170 160 150 140 130 120 110 100 90 80 70 60 50 40 30 20 10 ppm

Figure S13 a).

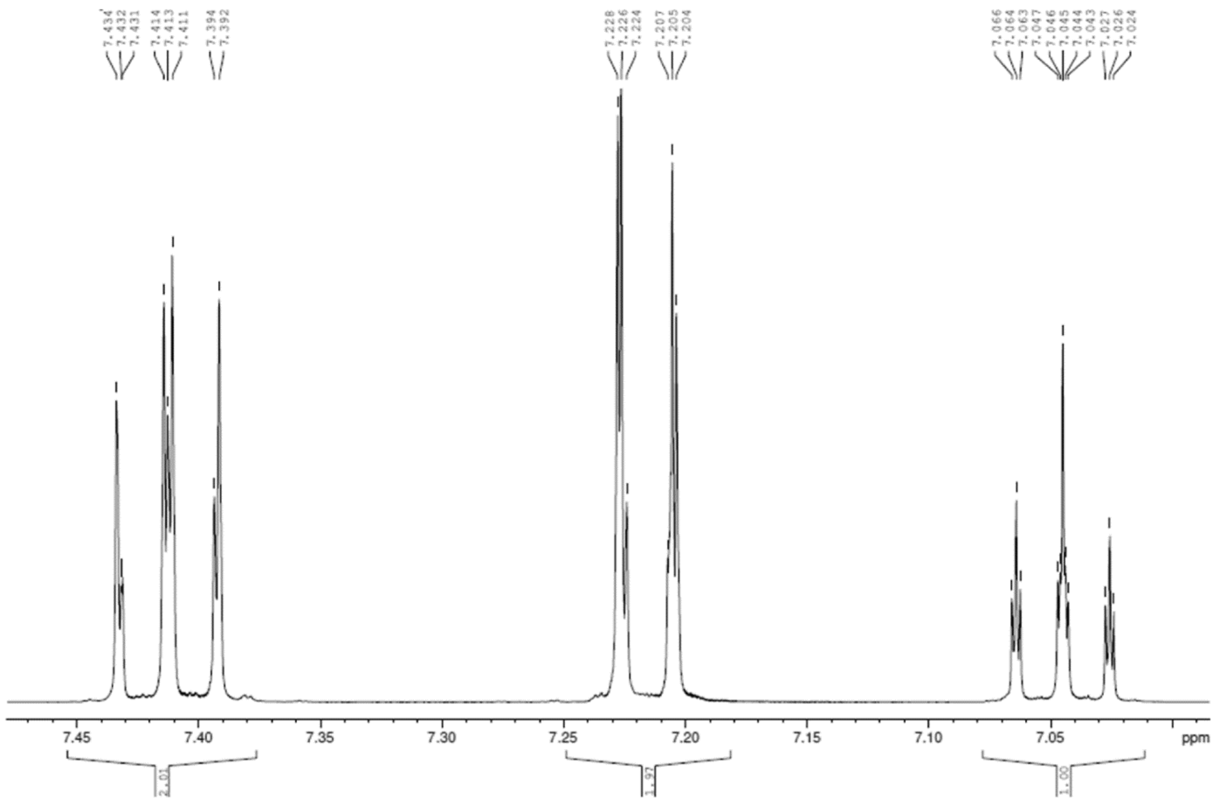

Figure S13 b).

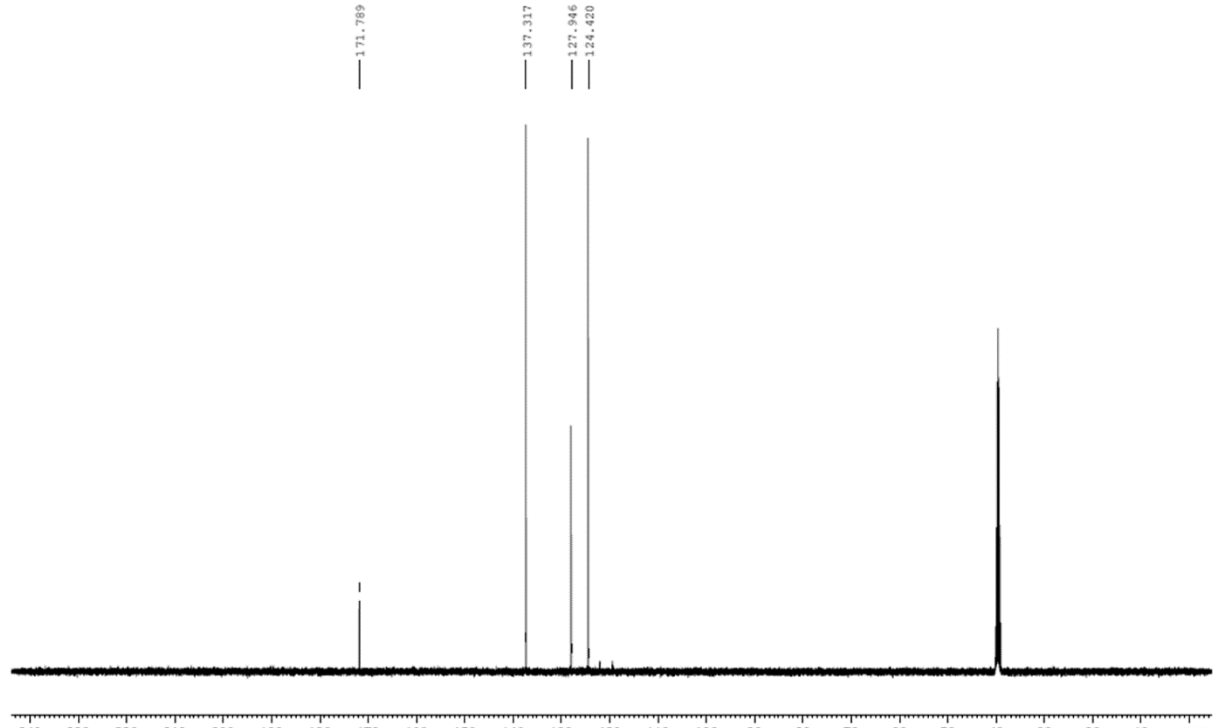

Figure S13 c).

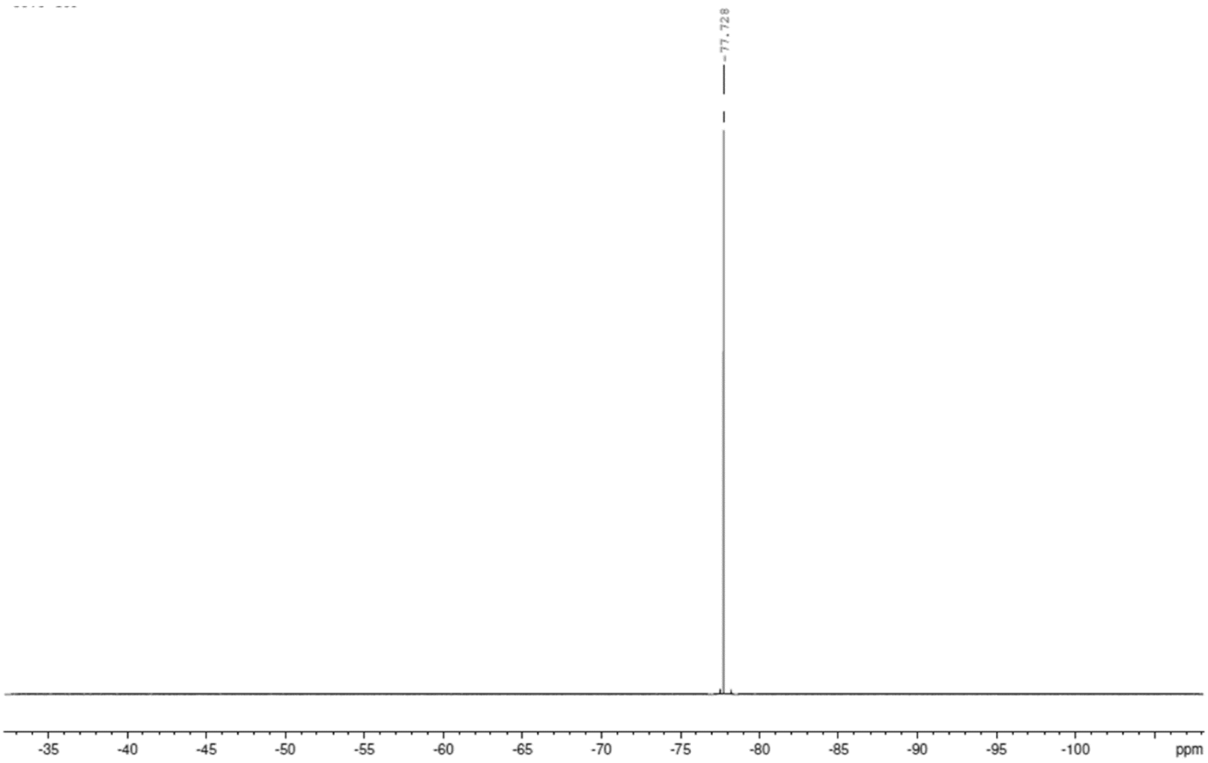

Figure S14 a).

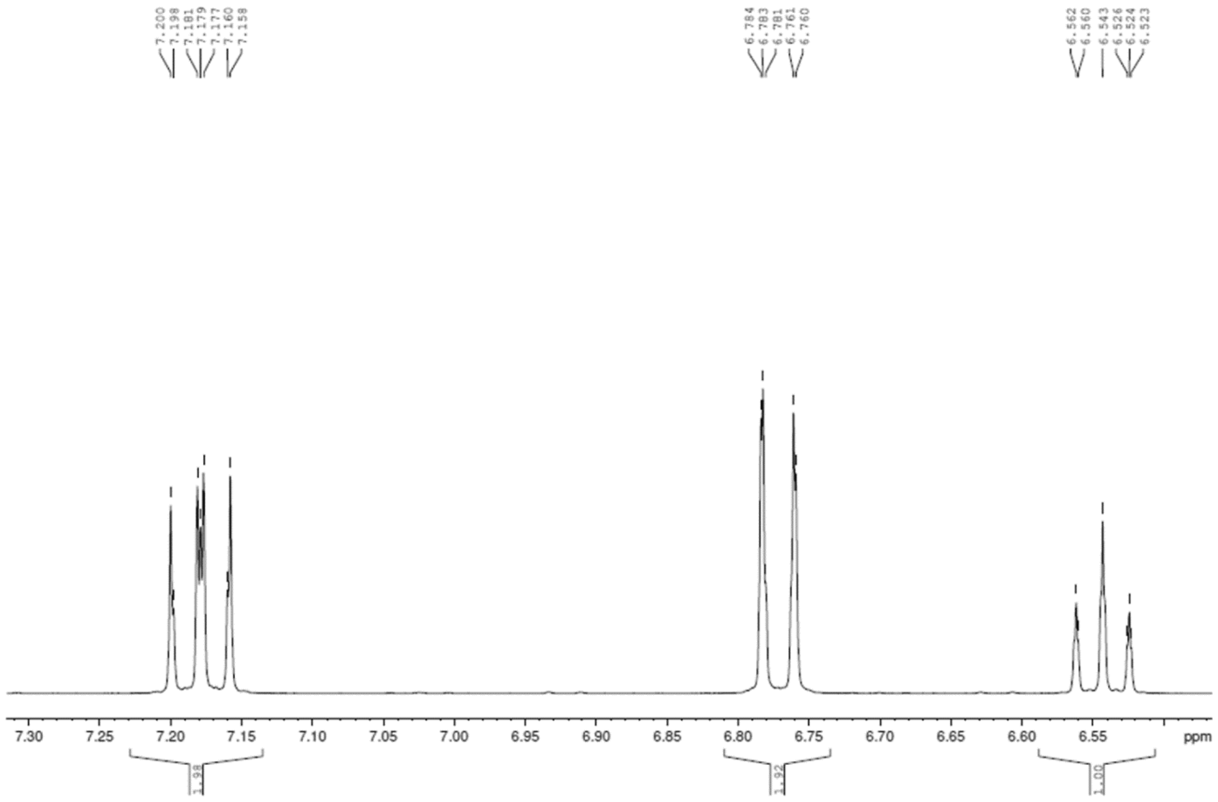

Figure S14 b).

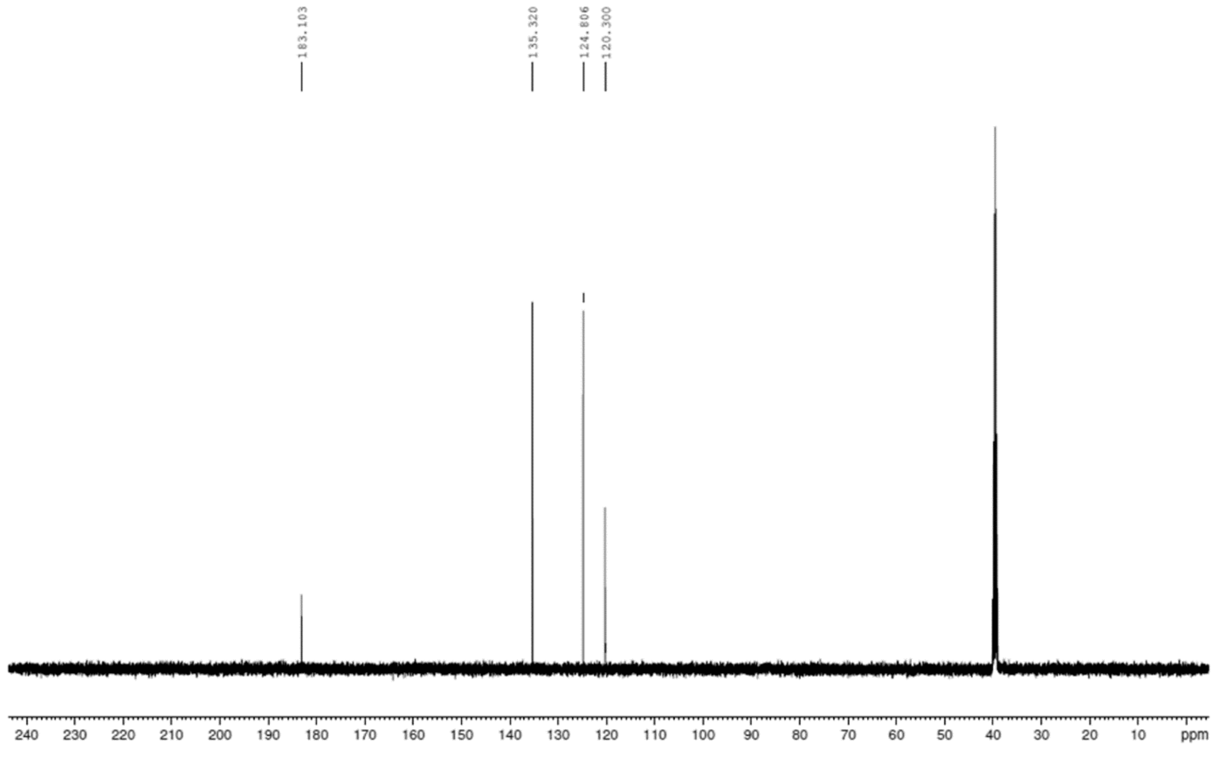

Figure S15 a).

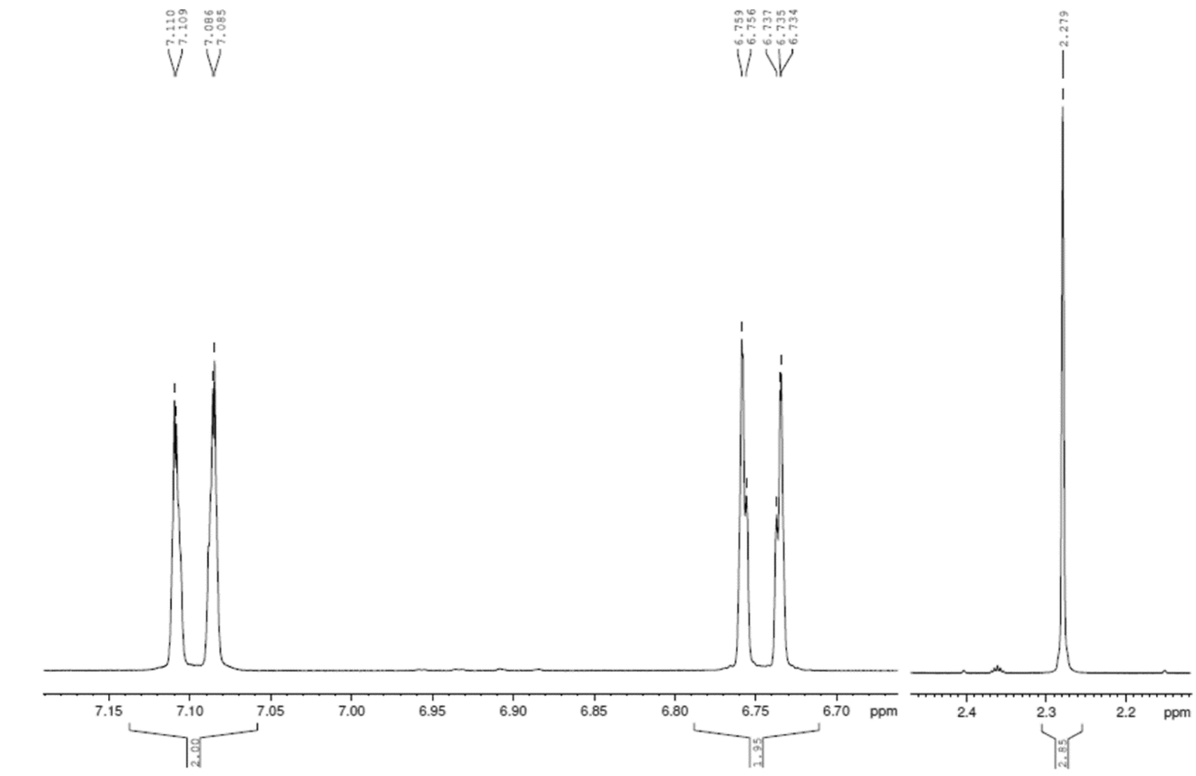

Figure S15 b).

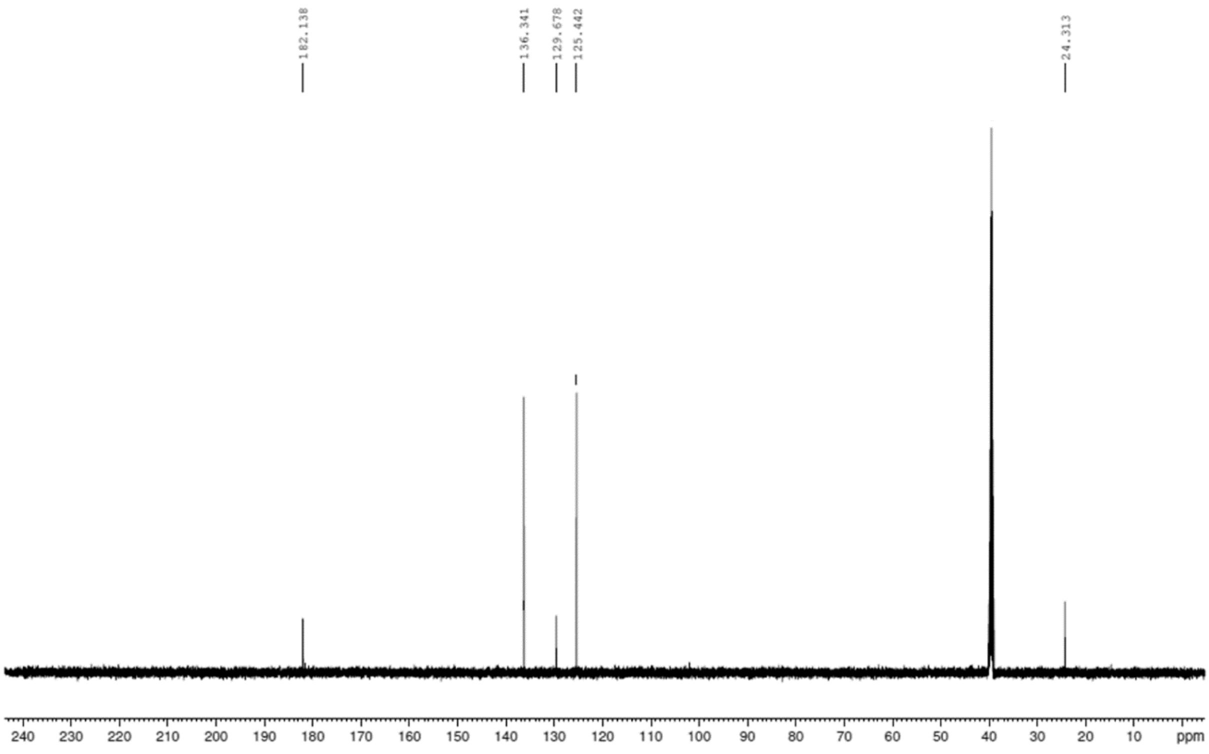

Figure S16 a).

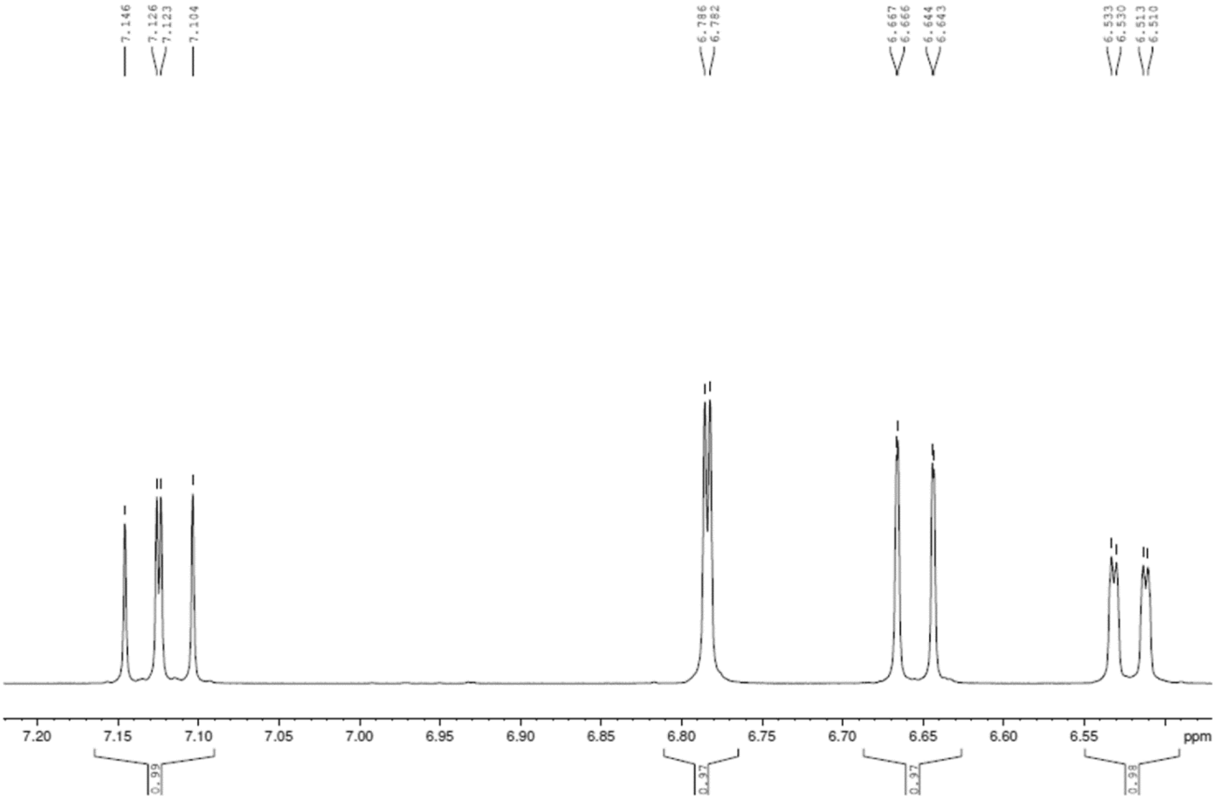
7.146
7.126
7.123
7.104
6.786
6.782
6.667
6.666
6.644
6.643
6.533
6.530
6.513
6.510
7.20 7.15 7.10 7.05 7.00 6.95 6.90 6.85 6.80 6.75 6.70 6.65 6.60 6.55 ppm
0.99
0.97
0.97
0.98

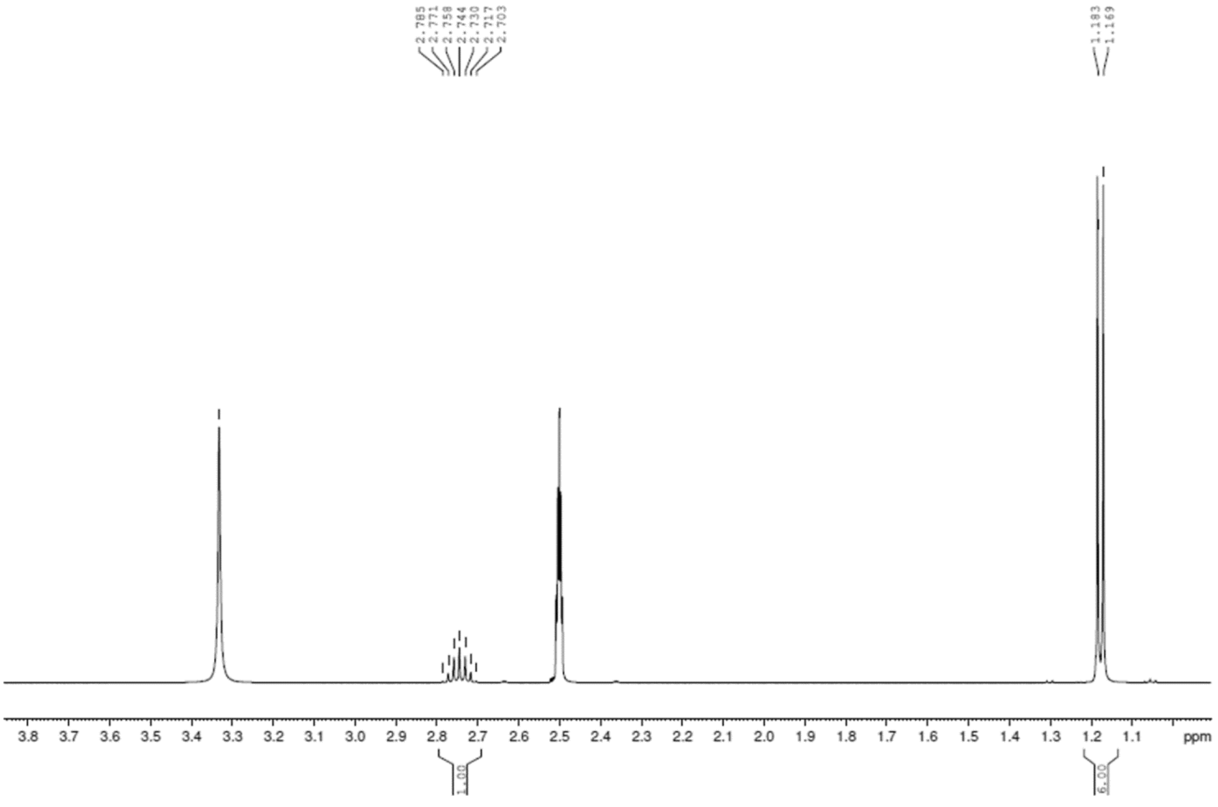
2.785
2.771
2.758
2.744
2.730
2.717
2.703
1.183
1.169
3.8 3.7 3.6 3.5 3.4 3.3 3.2 3.1 3.0 2.9 2.8 2.7 2.6 2.5 2.4 2.3 2.2 2.1 2.0 1.9 1.8 1.7 1.6 1.5 1.4 1.3 1.2 1.1 ppm
1.00
6.00

Figure S16 b).

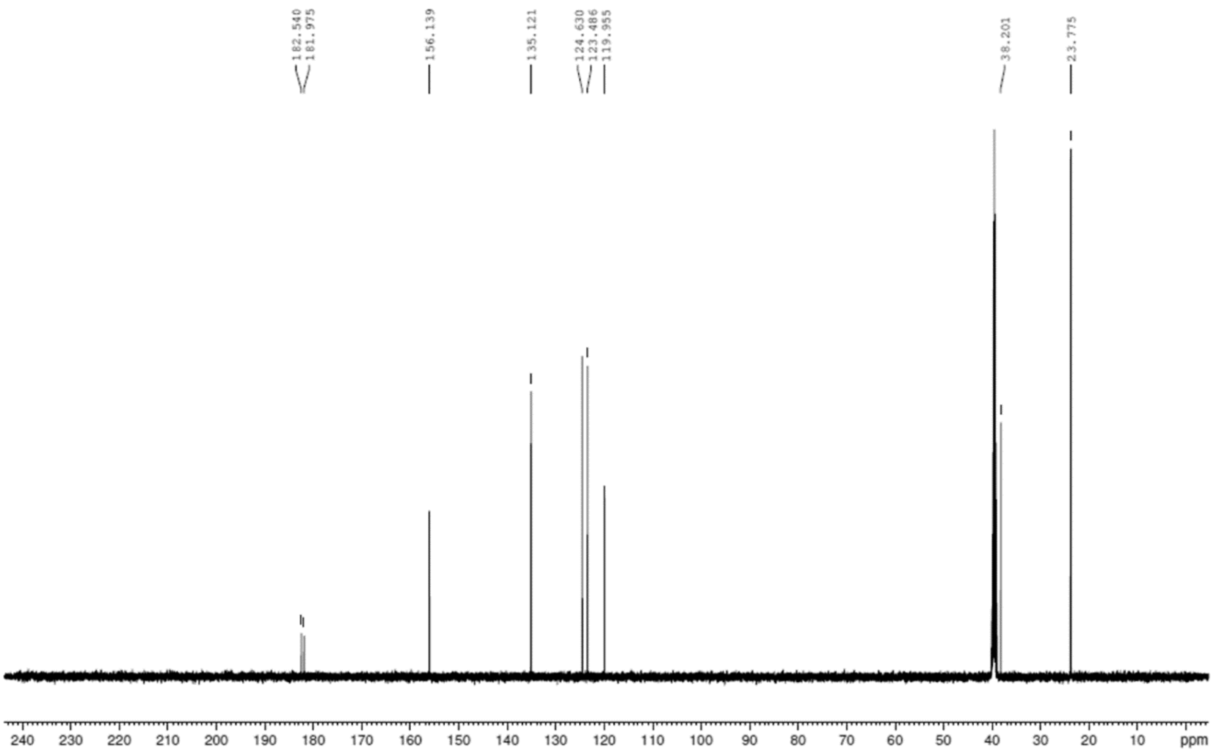
182.540
181.975
156.139
135.121
124.630
123.486
119.955
38.201
23.775
240 230 220 210 200 190 180 170 160 150 140 130 120 110 100 90 80 70 60 50 40 30 20 10 ppm

Figure S17 a).

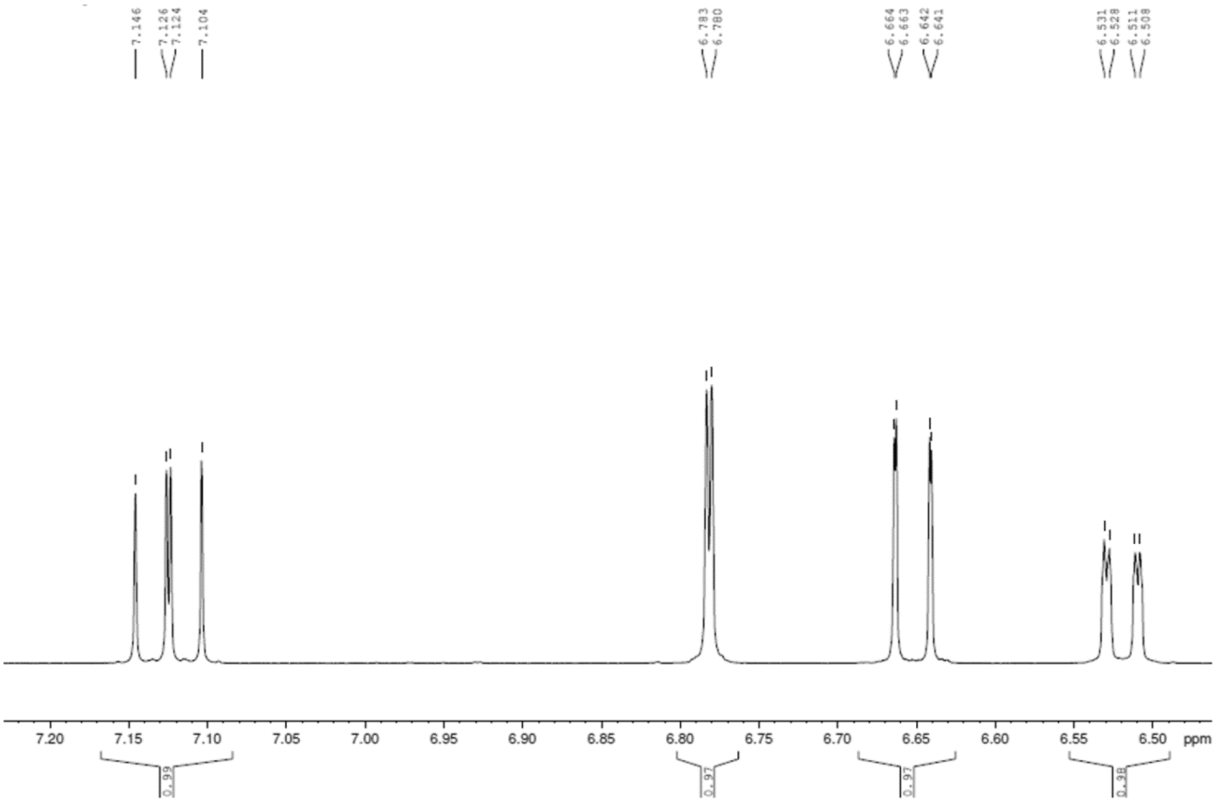
7.146
7.126
7.124
7.104
6.783
6.780
6.664
6.663
6.642
6.641
6.531
6.528
6.511
6.508
7.20 7.15 7.10 7.05 7.00 6.95 6.90 6.85 6.80 6.75 6.70 6.65 6.60 6.55 6.50 ppm
0.99
0.97
0.97
0.98

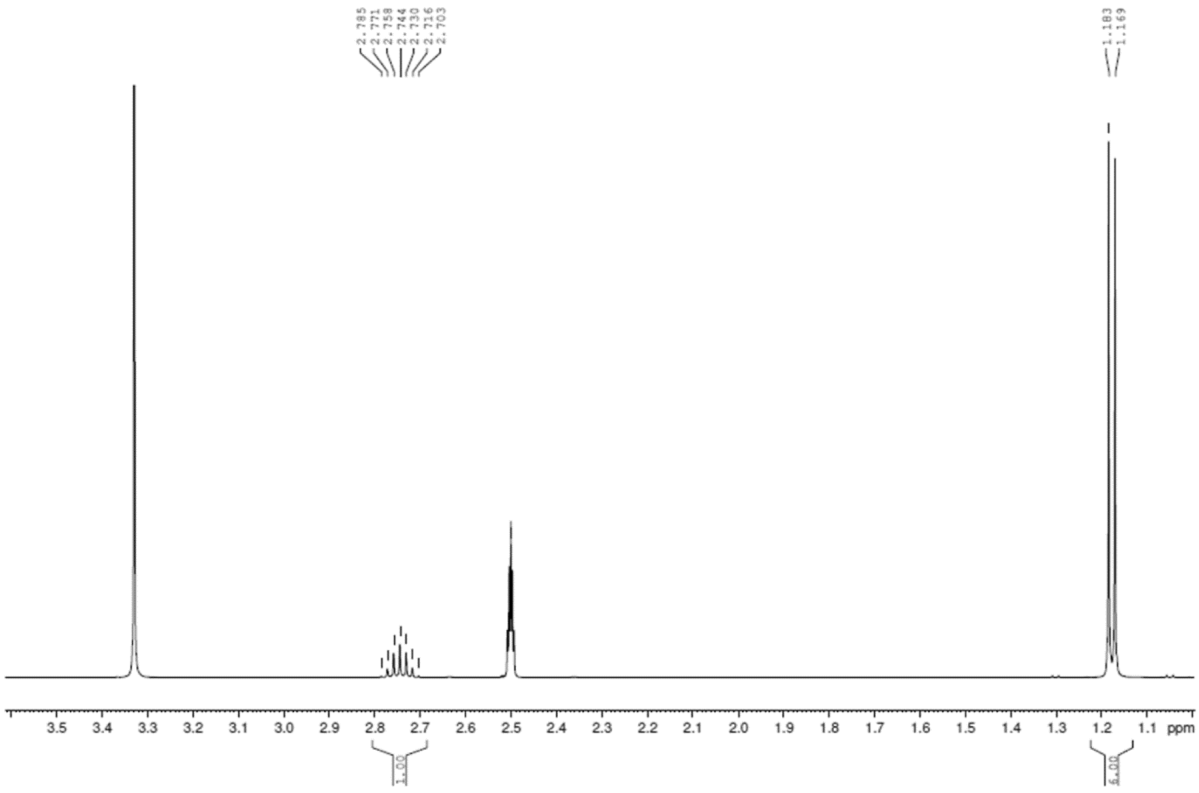
2.785
2.771
2.758
2.744
2.730
2.716
2.703
1.183
1.169
3.5 3.4 3.3 3.2 3.1 3.0 2.9 2.8 2.7 2.6 2.5 2.4 2.3 2.2 2.1 2.0 1.9 1.8 1.7 1.6 1.5 1.4 1.3 1.2 1.1 ppm
1.00
6.00

Figure S17 b).

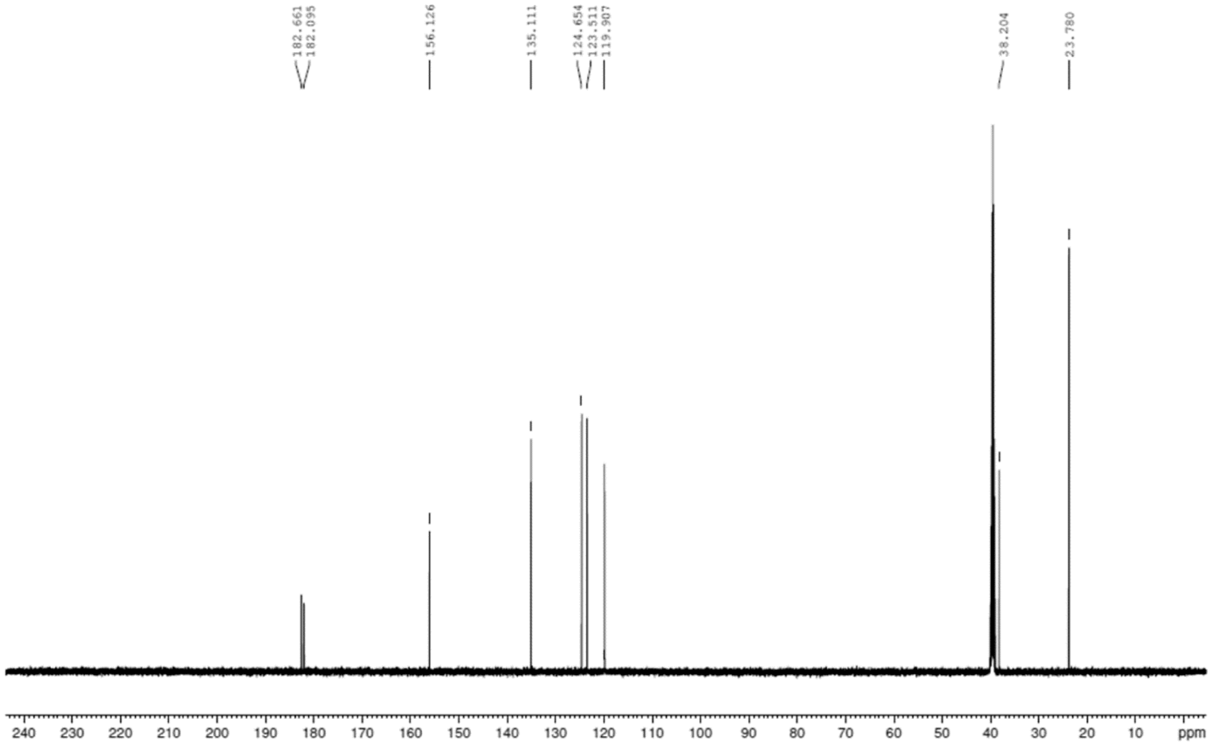

Figure S17 c).

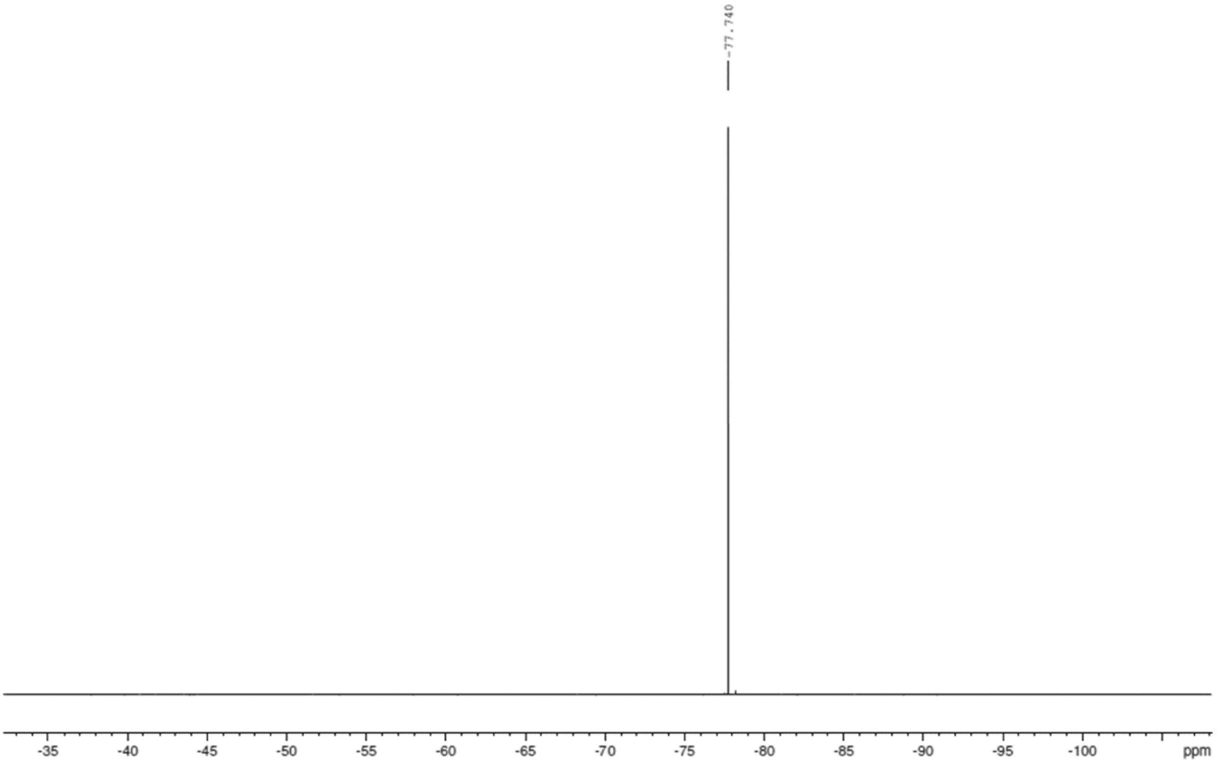

Figure S18.

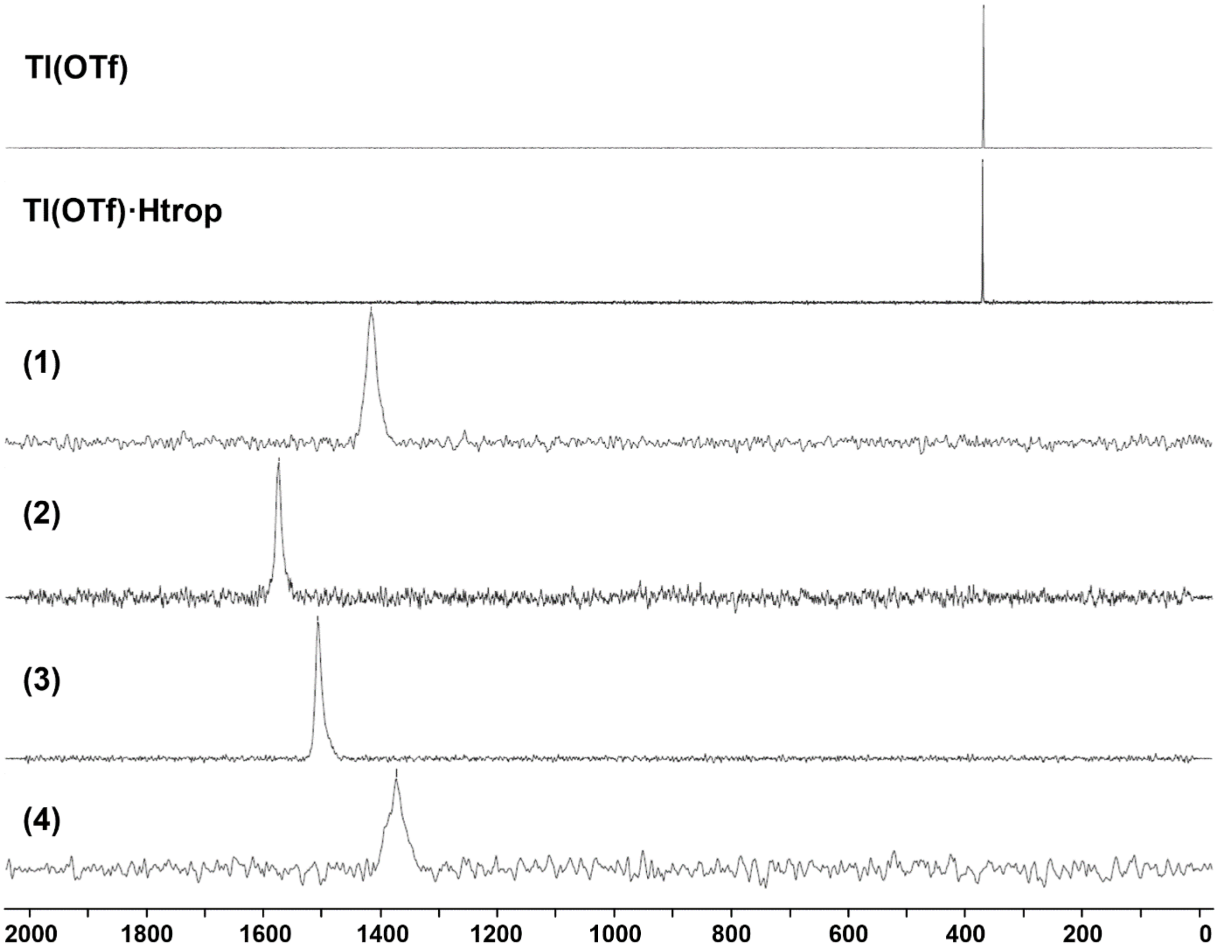
Tl(OTf)
Tl(OTf)·Htrop
(1)
(2)
(3)
(4)
2000
1800
1600
1400
1200
1000
800
600
400
200
0
